# Integrated photonic 3D tensor processing engine

**DOI:** 10.1038/s41377-026-02183-y

**Published:** 2026-03-06

**Authors:** Yue Wu, Ziheng Ni, Xin Li, Yuanxun Wang, Liangjun Lu, Jianping Chen, Linjie Zhou

**Affiliations:** 1 State Key Laboratory of Photonics and Communications, School of Integrated Circuits, Shanghai Jiao Tong University, Shanghai, 200240 China; 2 SJTU-Pinghu Institute of Intelligent Optoelectronics, Pinghu, 314200 China

**Keywords:** Integrated optics, Silicon photonics, Optoelectronic devices and components

## Abstract

Optical computing leverages high bandwidth, low latency, and power efficiency, which is considered as one of the most effective solutions for accelerating deep learning tasks. However, mainstream photonic hardware accelerators are primarily optimized for two-dimensional (2D) matrix-vector multiplications (MVMs). To implement three-dimensional (3D) convolutional neural networks (CNNs), high-order tensors must be reshaped in the electrical domain according to the size of the accelerators before computation, leading to extra memory usage and time overheads. Additionally, synchronization across multiple channels depends on external electronic clocks, which increases the complexity of the system. In this work, we propose an integrated photonic 3D tensor processing engine (3D-TPE) based on the interleaving modulation of time, wavelength, and space. Data caching, channel synchronization and computation are realized entirely within the optical domain, reducing memory and time usage, and simplifying the system. Optical caching and synchronization are achieved with an optical tunable delay line (OTDL) chip supporting versatile clock frequencies up to 200 GHz, and optical computing is accomplished with a dual-coupled micro-ring resonators (MRRs) based crossbar chip with a 3-dB passband width of 50 GHz. We verify the processing capabilities of the 3D-TPE at clock frequencies ranging from 10 GHz to 30 GHz and perform a proof-of-concept experiment for a LiDAR 3D point cloud image recognition task operating at 20 GHz, achieving a recognition accuracy of 97.06%. The proposed 3D-TPE is anticipated to facilitate high-order tensor convolutions, playing an important role in autonomous driving, healthcare, video analytics, virtual reality, etc.

## Abstract (web summary)

An integrated photonic 3D tensor processing engine enables 3D tensor computation, caching, and synchronization with tunable clock frequencies, experimentally achieving operation from 10 GHz to 30 GHz and 97.06% LiDAR point-cloud classification accuracy.

## Graphical abstract

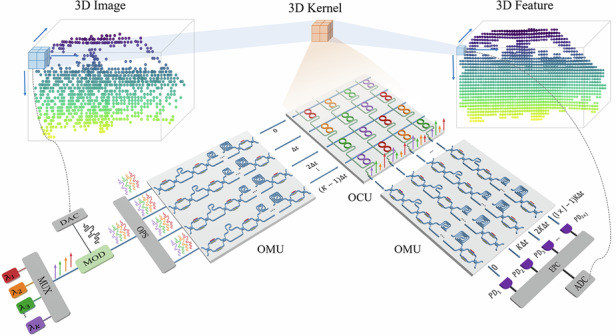

## Introduction

Deep learning-driven artificial intelligence (AI) has achieved significant breakthroughs in data-intensive tasks^1^. Convolutional neural networks, which extract data features through layers of convolutional filters, are among the most powerful tools in deep learning^2^. Propelled by the development of smart sensors^3,4^, information extraction now occurs across time, space, frequency, and other parameter spaces, forming high-order tensors^5^. While 2D CNNs process data on a 2D plane, 3D CNNs leverage 3D kernels to explore internal relationships within tensors across spatial and temporal dimensions^6^, playing a crucial role in 3D medical image segmentation^7–9^, video analysis^10,11^, autonomous driving^12,13^, and other fields^14–16^. However, the cubic increase in computation and memory overheads in the 3D CNNs presents significant challenges to hardware processing capabilities. With the exponential growth of AI models, high-speed and energy-efficient hardware accelerators are urgently desired^17,18^.

Electronic computing accelerators, such as Graphics Processing Units (GPUs)^19^, Tensor Processing Units (TPUs)^20^, memristor crossbar arrays^21^, and Field-Programmable Gate Arrays (FPGAs)^22^, have been extensively developed to meet computing power requirements. However, most of these hardware accelerators are optimized for 2D MVMs, which may not be optimal for accelerating 3D CNNs^23^. On the other hand, joule heating and operation bandwidth limitations inherent in electronic components hinder further improvements in computing speed^24^. Photonic processors mitigate data transfer bandwidth bottlenecks caused by capacitor charging and discharging processes in electronic processors, enabling processing bandwidths exceeding hundreds of gigahertz^25^. Moreover, they offer advantages of low latency, minimal power dissipation, and high degrees of modulation freedom across wavelengths, waveguide modes, polarization, time and space^26^. Therefore, photonic computing accelerators are promising candidates to address the information processing challenges of data-intensive applications.

**Fig. 1:**
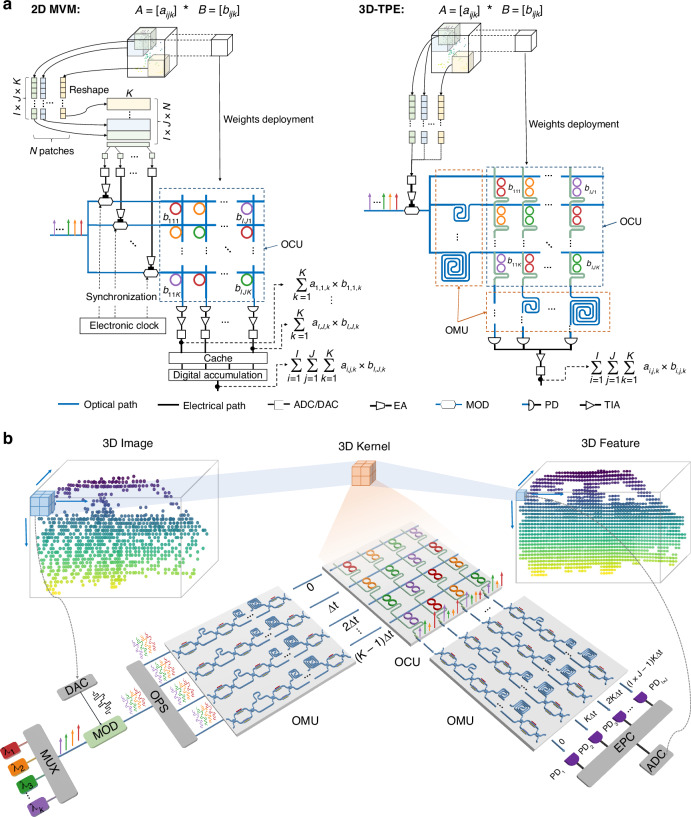
Concept of integrated 3D photonic processing engine. **a** Comparison between conventional 2D MVM photonic accelerators and the proposed 3D-TPE in 3D tensor convolutions. In 2D MVM accelerators (left), input data must be reshaped according to the size of 3D kernel in the physical implementation before being fed into the modulator array. Channel synchronization relies on an electronic clock module, and accumulation across different columns are performed in the digital domain. In contrast, the proposed 3D-TPE (right) enables data caching, channel synchronization, and computation entirely within the optical domain, significantly reducing system complexity. **b** Conceptual schematic of the proposed 3D-TPE. The OMU offers optical caching and channel synchronization, consists of on-chip tunable delay lines for adaptive clock frequency. The OCU is a dual-coupled-MRRs-based crossbar circuit for weighting and wavelength routing. ADC analog-to-digital converter, DAC digital-to-analog converter, EA electrical amplifier, MOD modulator, PD photodetector, TIA trans-impedance amplifier, OCU optical computing unit, OMU optical memory unit, MUX multiplexer, OPS optical power splitter, PD photodetector, EPC electrical power combiner

Various photonic tensor processors have been demonstrated. Utilizing singular value decomposition theory, 2D MVM operations based on Mach-Zehnder interferometer (MZI) mesh have been validated for CNN acceleration^27,28^, but the complexity of coherent optical phase tuning remains a significant challenge^29,30^. Photonic tensor processors based on wavelength division multiplexing (WDM) technology mitigate coherent phase errors by encoding data onto individual wavelengths^31–34^, which greatly improves parallel computing speeds. To further enhance parallelism, hybrid modulation combining WDM and radio frequency (RF) continuous wave signals^35^ and partial coherent optical modulation^36^, have been explored. Additionally, by utilizing optical delay lines or single-mode fibers, data caching has been performed in the optical domain^37–39^. Despite these remarkable achievements in photonic computing acceleration, the aforementioned schemes are primarily optimized for 2D MVMs. When performing 3D tensor convolutions, as illustrated in the “2D MVM” section of Fig. 1a, the 2D MVM hardware accelerators suffer from several limitations: (ⅰ) Input data must be reshaped according to the size of the 3D kernel in the physical implementation before being fed into the modulator array. The storage requirement for *N* patches is $N\times O(I\times J\times K)$, where $I,J,K$ are the dimensions of the 3D kernel. This process involves large-scale data reordering, leading to significant memory and time overheads. Since the computation must wait until the input data reshaping is completed, the bottleneck between the memory and the computation unit cannot be effectively broken. (ⅱ) Synchronization between multi-channel high-speed signals relies on external electronic clocks. As the size of photonic accelerators scales, system complexity increases dramatically, along with the increased number of high-speed modulators, electrical amplifiers (EAs), digital-to-analog converters (DACs), trans-impedance amplifiers (TIAs), and analog-to-digital converters (ADCs), leading to increased energy consumption and costs. (ⅲ) Due to the limited number of weighting elements (WEs) per column^31,40^, the 3D kernel convolution is decomposed into $I\times J$ vector multiplications in different columns and accumulated digitally. The storage usage for the temporary results is estimated to be $N\times O(I\times J)$. (ⅳ) Schemes with optical data caching are based on fixed delay lines or single-mode fibers, which cannot adapt to varying symbol rate requirements in time-sparse and time-dense application scenarios.

In this work, we propose an integrated photonic 3D tensor processing engine (3D-TPE) that introduces two optical memory units (OMUs) at the input and output ports of the optical computing unit (OCU), as illustrated in the “3D-TPE” part of Fig. 1a. Input data are sequentially loaded onto multi-wavelength optical carriers via a modulator and replicated across both the wavelength and spatial domains. The memory and time overheads associated with data reshaping process in conventional 2D MVM schemes is thereby eliminated. By introducing OTDLs based OMU chips, channel synchronization with tunable clock frequencies is realized entirely within the optical domain, eliminating the need for electronic clock modules. Through the wavelength routing configuration in the OCU chip, combined with tunable delay time in the OMUs, kernel convolutions are extended across different columns of the OCU chip. The 3D kernel convolution can be accomplished directly as input data flow across the entire system, without decomposition into smaller kernel convolutions, thus avoiding temporary data storage and digital accumulation in electrical domain. Only one modulator, one EA, one ADC, one TIA, and one DAC are required in the 3D-TPE, significantly reducing the number of high-speed electrical devices by orders of magnitude, thus simplifying the system and also energy saving. Based on the interleaving modulation of wavelength, time and space, data caching, channel synchronization and computation are performed entirely within the optical domain when performing high-order convolution. The OMU consists of an integrated eight-channel tunable delay line chip with a tuning resolution of 4.93 ps, providing adaptive clock frequencies up to ~200 GHz. The OCU is a dual-coupled-MRRs crossbar chip for parallel multiplication computation via optical intensity modulation. Compared to single-MRR-based WEs used in conventional schemes, the dual-coupled-MRRs WE exhibits a flatter spectral response and a broader optical bandwidth, minimizing signal distortion at high symbol rates and enhancing resistance to laser wavelength shifts. In proof-of-concept experiments, four-channel matrix multiplication operations at clock frequencies ranging from 10 GHz to 30 GHz were demonstrated. A LiDAR 3D point cloud image recognition task was also performed at a symbol rate of 20 Gbaud, achieving a recognition accuracy of 97.06%, which is comparable to digital results. The proposed integrated photonic 3D-TPE demonstrates significant potential for general-purpose 3D tensor convolutions and 3D CNN acceleration, which is believed to play an important role in autonomous driving, real-time video analysis, 3D medical image processing, and other applications.

## Results

### Processing flow of the integrated photonic 3D-TPE

Figure 1b illustrates the schematic of the proposed integrated photonic 3D-TPE and its working principle for 3D kernel convolutions on a 3D point cloud image. A 3D convolutional kernel performs 3D kernel convolutions on the point cloud image within its coverage area, generating a 3D convolutional feature map by sliding the convolutional kernel in each of the three spatial directions. In the optical implementation, input image data is sequentially loaded onto the modulator and replicated across both wavelength and spatial dimensions. After passing through the OTDL array (marked as OMU), the delayed replicas are weighted by the dual-coupled-MRRs crossbar circuit (marked as OCU), where the weights of the 3D kernel are deployed. The outputs of the OCU are fed into another OMU and converted into photocurrents by photodetectors (PDs). Finally, the outputs of the PDs are summed by an electrical power combiner, and the 3D feature map is obtained by sampling the output waveforms.

**Fig. 2:**
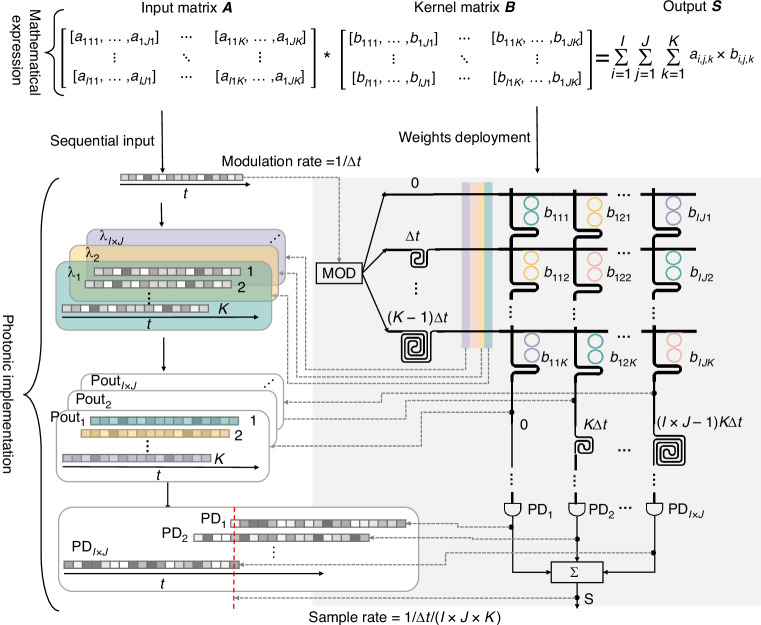
Processing flow of the 3D-TPE. The input 3D matrix ***A***, with a size of ($$I,J,K)$$, is sequentially modulated onto multi-wavelength carriers by an intensity modulator. The 3D kernel matrix ***B***, with a size of ($$I,J,K)$$, is deployed on the dual-coupled-MRRs based crossbar circuit. The 3D convolution operation is accomplished by controlling the time delay intervals between channels as well as the coding process

The mathematical expression and optical implementation of the 3D convolution are illustrated in Fig. 2. A 3D matrix ***A***, with a size of $\left(I,J,K\right)$, where $I\times J\ge K$, is sequentially modulated onto $I\times J$ individual wavelength carriers by an intensity modulator with a symbol duration of $\Delta t$. After optical intensity modulation, the signals are evenly divided into *K* paths, accomplishing $I\times J\times K$ replications across both wavelength and spatial dimensions. The replicas are then fed into an OMU chip. By selecting different optical paths within the OTDLs, time delay ranging from $0$ to $(K-1)\,\triangle t$ with equal time intervals of $\Delta t$ (equal to the symbol duration) for *K* paths are introduced. The delayed multi-wavelength signals are then routed into the OCU chip via horizontal bus waveguides and subsequently combined into vertical bus waveguides through the filtering effect of the MRRs. The OCU is structured with $K$ rows and$\,I\times J$ columns, with 3D matrix ***B*** deployed on it. The dual-coupled-MRRs structure serves as the basic WE, enabling wavelength filtering and weighting functions simultaneously. Each WE processes a single wavelength channel, and there is no WE working on the same wavelength in any row or column, thereby preventing coherent interference. After being weighted by the WEs, the outputs of the OCU chip are fed into another OMU with time intervals of $K\triangle t$ for $I\times J$ OTDLs. Thus, all weighting channels have equal time intervals of $\triangle t$ with a total delay time ranging from 0 to $(I\times J\times K-1)\triangle t$. PD arrays and an electrical power combiner are used to perform the accumulation. Finally, the 3D convolutions are obtained by sampling the output waveform at a time interval of $(I\times J\times K)\triangle t$. Furthermore, by utilizing the spectral repetition properties in various resonance orders of the MRR, the outputs of the crossbar circuit can be combined by an optical power combiner and accumulated by a PD, as described in supplementary note 1 for more details.

### Dual-coupled-MRRs based crossbar OCU

**Fig. 3:**
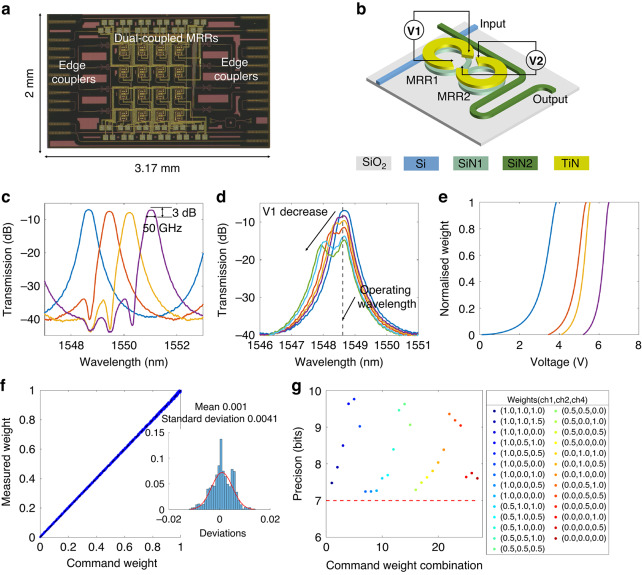
3D dual-coupled-MRRs crossbar OCU. **a** Microscope image of the fabricated 4×4 crossbar chip. **b** Schematic structure of the 3D dual-coupled-MRRs on a multilayer Si_3_N_4_-on-SOI platform. The transmission spectrum can be tuned by adjusting voltages applied to MRRs. **c** Measured transmission spectra of four WEs. **d** Measured transmission spectra under different weights. The weight tuning is accomplished by varying the voltage applied to MRR1 while keeping MRR2 fixed. **e** Normalized weight-voltage relationship curves. **f** Scatterplot between command weights and measured weights for 27 weight combinations. The inset shows the error distribution with a standard deviation of 0.0041 and a mean error of 0.001. **g** Weight accuracies corresponding to each of the 27 weight combinations (shown in the legend), respectively

The OCU used in this work is a 4×4 dual-coupled-MRRs crossbar chip, fabricated on a multilayer Si_3_N_4_-on-SOI platform. Figure 3a shows the microscope image of the fabricated chip with a footprint of 2 mm × 3.17 mm. The OCU consists of 16 WEs, each implemented as a 3D dual-coupled-MRRs structure, as depicted in Fig. 3b. The dual-coupled MRRs are constructed on the middle Si_3_N_4_ layer, which are coupled to the bottom silicon waveguide and the top Si_3_N_4_ waveguide, respectively, forming an add-drop filter configuration. The lower refractive index contrast (~0.56) and lower thermo-optic coefficient of the Si_3_N_4_ waveguide make the MRRs less sensitive to fabrication deviations and ambient temperature variations compared to silicon-based MRRs^41^. To minimize chip loss and loss non-uniformity among weighting channels, an optimized 3D waveguide crossing constructed by the bottom silicon waveguide and top Si_3_N_4_ waveguide is introduced. More details of the chip design can be found in our previous work^42^.

In the weight tuning process, voltages (V1 and V2) were first applied to the two MRRs within each WE to align their resonances to the operating wavelength. Figure 3c presents the measured transmission spectra of four dual-coupled-MRRs WEs around the operating wavelengths, which has a channel spacing of 97 GHz. The transmission spectrum of each WE exhibits a box-like shape, with an optical 3-dB bandwidth of approximately 50 GHz and crosstalk below -25 dB. Compared with single-MRR-based WEs, the dual-coupled-MRRs WEs can offer a broader optical bandwidth to mitigate signal distortion in high-speed signal processing, lower crosstalk from adjacent channels, and flatter spectral characteristics to minimize weighting errors (supplementary note 2). After resonance wavelength alignment, the voltage (V2) on one MRR was fixed, while the voltage (V1) applied on the other MRR was varied. This modulation alters the optical power at the operating wavelength, and the corresponding transmission spectrum as a function of the sweeping voltage is depicted in Fig. 3d. Finally, by normalizing the optical power at the operating wavelengths to 0 and 1 (supplementary note 2), the relationship between weight values and the applied voltages (V1) was obtained. Figure 3e presents the weight-voltage (W-V) relationships across four WEs. To be noted, the W-V curve of each WE was measured when the weights of adjacent WEs were fixed at 0.5. Since all the dual-coupled-MRRs WEs share identical design parameters, these WEs exhibit nearly identical initial resonant wavelengths, benefiting from the high fabrication tolerance of the 3D dual-coupled-MRRs design. In the 3D-TPE system, each WE must be tuned to its designated operating wavelength before weight deployment. As a result, the applied voltage range required for weight tuning varies for each WE, as shown in Fig. 3e.

The influence of thermal crosstalk on weighting accuracy was evaluated by measuring the weights of a single WE in various weights conditions of adjacent WEs. In the experiment, 50 randomly generated weight values were applied to the third WE in a column, while three weight values, randomly combined from 0, 0.5, and 1, were assigned to the remaining three WEs. Therefore, 27 weight combinations, each with 50 randomly generated weight values, were measured. Figure 3f shows a scatterplot of the commanded weights versus measured weights across the total 1350 measurements. The inset illustrates weighting errors, with a mean value of 0.001 and a standard deviation of 0.0041. Furthermore, weighting accuracies across the 27 weight combinations were compared, as shown in Fig. 3g, demonstrating equivalent weighting accuracies exceeding 7 bits for all combinations. Given that the W-V relationships were calibrated with weights of adjacent WEs set to 0.5, the equivalent weighting accuracy reached approximately 9.7 bits under initial conditions. The experimental results demonstrate that the 3D OCU chip, fabricated on a multilayer Si_3_N_4_-on-SOI platform, can achieve high-precision arbitrary weight tuning without complicated algorithms or feedback control schemes^39,43–45^ due to their ambient temperature insensitivity and flat-top spectral characteristics.

To further verify the parallel computing capabilities of the OCU chip, a 2D CNN experiment for the MNIST handwritten digit recognition task was constructed. Experimental results indicate that the recognition performance of the OCU chip is comparable to that of a digital computer. More details about the experiment can be found in supplementary note 3.

### Tensor processing with tunable clock frequencies

**Fig. 4:**
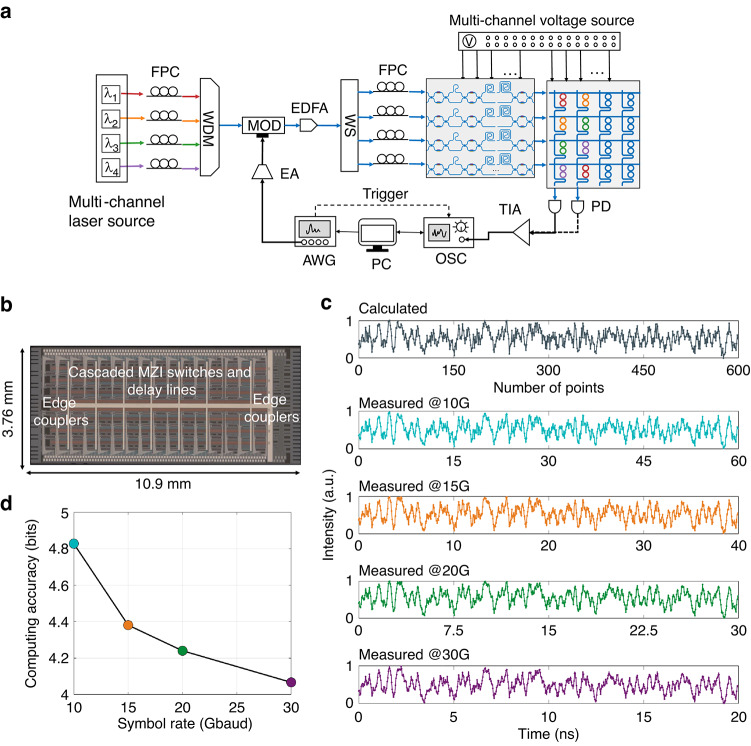
OTDLs for tunable symbol rates. **a** Experimental setup for 4-channel matrix multiplication. **b** Microscope image of the fabricated optical OTDLs. **c** Comparison of measured and calculated waveforms for four-channel matrix multiplication at different symbol rates. **d** Computing accuracies at various symbol rates. FPC fiber polarization controller, WDM wavelength division multiplexer, EDFA erbium-doped fiber amplifier, WS wave-shaper, AWG arbitrary waveform generator, OSC oscilloscope

The experimental setup for parallel four-channel matrix multiplication at multiple tunable clock frequencies is illustrated in Fig. 4a. Four individual wavelengths were combined via a WDM and subsequently fed into the 3D-TPE. The input data were modulated onto the optical carriers via a high-speed modulator driven by an arbitrary waveform generator (AWG) at a symbol rate of $1/\Delta t$, and then split into four before entering the OMU chip. The OTDLs within the OMU chip were configured with time intervals of $\Delta t$ to enable data caching and channel synchronization. The delayed replicas were then weighted by a column of four WEs within the OCU chip. The output was monitored by a PD and recorded by an oscilloscope. Rate-adaptive optical computing at variable clock frequencies were realized by varying the symbol rates of the input waveform and the corresponding time intervals between OTDLs. More details of the experimental setup can be found in “Materials and methods”.

The OMU chip is the key device enabling task-adapted clock frequency reconfiguration in the 3D-TPE. Figure 4b shows a microscope image of the OMU chip used in this study, which was fabricated on the SOI platform with a chip size of 3.76 mm × 10.9 mm. It consists of 8 identical OTDLs, each comprising 7 cascaded MZI switches connected by two delay waveguides in between. The two delay waveguides are designed with different lengths, resulting in different group delays. The differential group delay of the *n*^th^ (*n* = 1, 2, …,6) delay waveguide pair is 2^*n*-1^*δt*, where *δt* represents the delay resolution. By controlling the states of the cascaded MZIs to alter optical paths, the group delay can be tuned from 0 to 63δt. Experimental results indicate that the chip achieves a delay resolution of 4.93 ps, thus a maximum delay time of 310.59 ps is enabled. More details regarding the design and testing of the delay line can be found in supplementary note 4.

Before performing the computations, channel synchronization calibration was taken with a time interval of 50 ps between four computing channels (supplementary note 5). Computing accuracy was evaluated using 120,000 randomly generated data points ranging from 0 to 1 at symbol rates of 10 GBaud, 15 GBaud, 20 GBaud, and 30 GBaud. The four WEs were configured with fixed weight values of $\left[0.90,0.54,0.61,0.76\right]$. Figure 4c presents a segment of the output waveforms recorded by a real-time OSC at various symbol rates, which closely align with the digital calculated results. Weighting errors between the measured and calculated results were analyzed, with the equivalent bits of the standard deviations ranging from 4.1 bits to 4.8 bits, as shown in Fig. 4e. As the symbol rate increases, computing accuracy decreases slightly due to the limited analog bandwidth of AWG (25 GHz) and increased signal distortion and transmission noise at higher modulation speeds. The computing accuracy can be further improved by monolithically integrating all key components to reduce system optical loss and enhance stability, as well as by implementing weight control algorithms^43^ and high-speed signal pre-compensation techniques^46^.

### 3D LiDAR point cloud image recognition

**Fig. 5:**
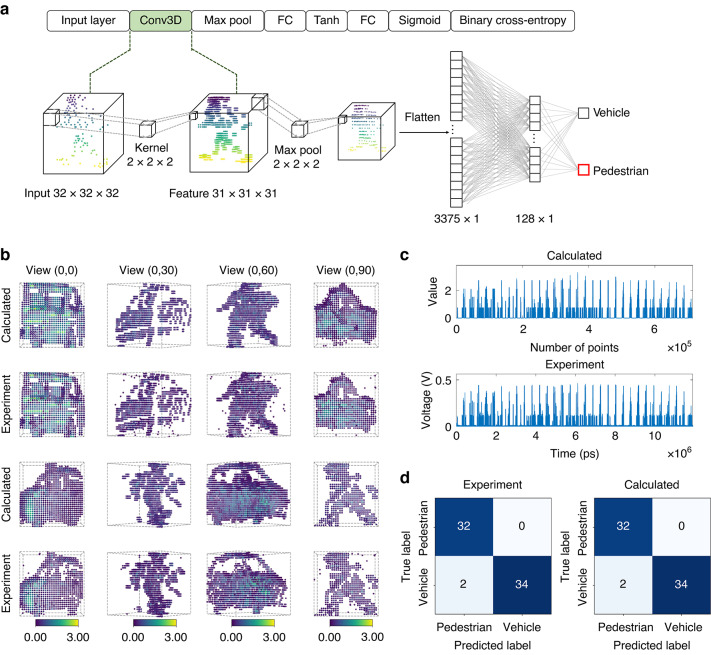
3D point cloud recognition with a 3D CNN. **a** The designed 3D CNN architecture consists of a convolution layer, a max pooling layer, and two fully connected layers. The 3D convolution (highlighted with a green background) is implemented on the 3D-TPE. **b** Calculated and experiment 3D feature maps at different viewing angles. Colors indicate the intensity values of point clouds, providing information about surface characteristics like reflectivity and material. **c** Comparison between the digitally calculated waveform and the experimental waveform. **d** Confusion matrix of calculated and experimental results, with an accuracy of 97.06%

In recent years, deep learning for point clouds has received increasing attention owing to its broad applications in autonomous driving, computer vision, virtual reality, and other domains^47^. To further validate the proposed 3D-TPE, a two-class 3D LiDAR point cloud image recognition experiment was conducted to distinguish pedestrians and vehicles at a symbol modulation rate of 20 GBaud. The dataset used in this study is the Sydney urban objects dataset^48^, with cases acquired via a commercial LiDAR. The dataset contains 26 object classes with an uneven distribution. To balance the data for recognition, the bus, car, truck, and van classes were merged into a vehicle class, while the pedestrian class remained unchanged. Among the 340 available cases, 80% were randomly selected for training, and 20% were used for testing. Figure 5a illustrates the architecture of the designed 3D CNN, which consists of a convolutional layer with a 3D kernel, a max-pooling layer, and two fully connected layers, similar to the well-known VoxNet^13^. The network was pre-trained on a digital computer, with the convolutional kernel weights constrained to the positive real data domain. During image inference, the 3D kernel convolution (Conv3D) was executed on the 3D-TPE, while the remaining computations were performed on a digital computer.

The experimental setup is shown in Fig. 4a. 3D point cloud images, with a size of 32×32×32, were sequentially mapped to analog signals and transmitted to a high-speed modulator operating at a symbol rate of 20 GBaud. After being split into four paths by a WS, the modulated signals were delayed at 50 ps intervals using four OTDLs within the OMU chip. The 3D kernel, with a size of 2×2×2, was deployed on the OCU chip. As the delayed signals flowed through the OCU chip, matrix multiplication in a column of four WEs was performed. To construct eight weight values, the four WEs were reused twice to leverage two waveforms. A real-time OSC was used to recorded the waveforms, while the time delay and accumulation between them were processed digitally. Feature map data points were extracted by down sampling the real-time waveforms at a sample rate of 20/8 = 2.5 GSa s^-1^. Figure 5b compares the calculated and experimentally measured 3D feature maps at various viewing angles. Across different perspectives of pedestrians and vehicles, the 3D-TPE effectively captures key features, with minor discrepancies primarily attributed to signal distortion and noise interference during high-speed transmission. Figure 5c presents the real-time experimental waveform, which closely aligns the theoretical one. More experimental waveform comparisons can be found in supplementary note 6. Figure 5d displays the confusion matrix of the 3D point cloud recognition task, where the numbers in the upper left and lower right indicate correct classifications. The experimental recognition accuracy reaches 97.06%, which is equivalent to that of a digital computer. This experiment demonstrates the potential of the proposed 3D-TPE for 3D tensor convolution applications.

## Discussion

To address the limitations of 2D MVM accelerators in high-order data processing, a novel 3D tensor processing engine has been proposed. The 3D-TPE eliminates the memory and time overheads associated with the data reshaping process in conventional 2D MVM accelerators. Channel synchronization is achieved entirely in the optical domain via OTDLs within the OMU chip, eliminating the need for electrical clock modules. By configuring two OMUs alongside the OCU, convolution operations are extended across different columns of OCU chip, high-order convolutions can be executed directly as input data flow across the entire system without decomposing them into smaller kernel convolutions, thereby avoiding external electronic peripherals for temporary data storage and digital accumulation. The interleaved modulation of wavelength, time, and spatial domains enables data caching, channel synchronization and computation performed entirely within the optical domain. In the OMU, the adaptive OTDLs guarantee flexible system clock frequencies for various applications. In the OCU, dual-coupled-MRRs WEs fabricated on the multilayer Si_3_N_4_-on-SOI platform were proposed, which alleviate the bandwidth limitations and ambient temperature sensitivity compared to Si-based single MRR WEs. The multilayer ${{\rm{Si}}}_{3}{{\rm{N}}}_{4}$-on-SOI platform is a promising candidate for the 3D-TPE due to its mature integration processes for scalable manufacturing. Additionally, it enables minimized on-chip insertion loss and improved loss uniformity between channels of the OCU chip by utilizing optimized 3D waveguide crossing. For further integration, the integration with III–V materials (such as GaAs, InP, or AlGaAs) is expected. Using the advanced heterogeneous integration technology, III–V materials offer significant advantages in terms of laser sources, nonlinearities, and higher efficiency for photonic computing^49^. This combination could support both high-performance light sources and nonlinear functions, enabling more complex operations and accelerating optical neural networks. In addition, kerr optical frequency combs^50^, offering rich wavelength sources and compatibility with silicon-based fabrication process, although are constrained by limited output power, with the development of on-chip optical amplifiers^51^, are another promising candidates for future on-chip laser sources.

To further characterize the performance of the 3D-TPE, a computing energy efficiency estimation was taken (supplementary note 7). Enabled by the OTDLs, modulation rates from 10 Gbaud to 30 Gbaud have been experimentally verified, achieving a high throughput of up to 4×4×2×30 GOPS = 0.96 TOPS. The throughput is expected to further increase with the size scaling of the OMU and the OCU chips, as well as higher processing rates. In the 3D-TPE, the reduced number of modulators, EAs, TIAs, ADCs, and DACs significantly decreases system complexity and power consumption, resulting in a computing power efficiency of 0.3 TOPS W^-1^. Additionally, by integrating non-volatile phase-change materials (PCMs)^52^, the power required for maintaining the weight and optical delay states can be neglected. Currently, the core components of 3D-TPE are individually packaged, leading to unexpected coupling losses between devices (supplementary note 8). With advancements in photonic monolithic integration technology, the OCU chip, the OMU chip, modulator, and PDs can be monolithically integrated on a single chip, significantly reducing system footprint, system insertion loss, channel-to-channel loss variations, and enhancing system stability. However, after monolithic integration, thermal crosstalk between devices must be carefully managed, particularly for temperature-sensitive components. The estimated footprint of the integrated scheme is approximately 39.2 mm^2^, resulting in a computing density of 0.96/39.2 = 0.0245 TOPSmm^-2^ (supplementary note 9). Through delay-line optimization^53^ and the introduction of hybrid-integrated Si–PCM phase shifters^52^, the OMU chip footprint can be further reduced, thereby enhancing the computing density. Furthermore, the system scale can be expanded up to 121 WEs, operating at clock frequencies ranging from 10 GHz to 200 GHz, without significant accuracy degradation due to system losses (supplementary note 10).

In conclusion, we have proposed a 3D-TPE scheme consisting of two OMUs and one OCU, enabling data caching, channel synchronization and computation entirely within the optical domain. Compared with conventional 2D MVM schemes, the proposed approach significantly reduces memory usage, processing time, and overall system complexity during high-order kernel convolution tasks. The OMU, based on OTDLs, supports adaptive clock frequencies up to 200 GHz for diverse application requirements. The OCU, implemented using a dual-coupled-MRRs crossbar circuit, has a large 3 dB optical bandwidth of 50 GHz. Weighting accuracies exceeding 7 bits have been achieved at various weight combinations of adjacent WEs using a simple look-up-table method. In proof-of-concept experiments, the processing capability of the 3D-TPE has been demonstrated at modulation rates ranging from 10 GBaud to 30 GBaud. Additionally, a binary classification task of 3D LiDAR point cloud images was performed at a modulation rate of 20 GBaud, achieving a recognition accuracy of 97.06%, which is comparable to that of a digital computer. The proposed integrated photonic 3D-TPE holds significant promise for applications in autonomous driving, video analysis, medical imaging analysis, and other fields.

## Materials and methods

### Details for experimental setup

In the experiment, four individual continuous wave lasers (OVLINK TSP-1000) with a channel spacing of 100 GHz were combined using a WDM (Oplead 4CH-DWDM) and then transmitted into a high-speed modulator (MXAN-LN-40). The input digital signals were converted into analog electrical signals with a symbol duration of $\triangle t$ by an AWG (Keysight M8195A), and then amplified by an electric amplifier (SHF S807) before being applied to the modulator. After passing through an EDFA (Amonics AEDFA-23-B-FA) to compensate for optical insertion loss, the modulated optical carries were equally divided into four channels by a wave-shaper (Coherent Wave-shaper 4000B) and transmitted into four OTDLs within the OMU chip. A wave-shaper was employed instead of simple power splitter due to the fact that the wave-shaper offers programmable controls that allow individual power switching for each output, which benefits clock synchronization between computational channels. A multi-channel voltage source was used to provide voltages to the OMU chip and the OCU chip. The OMU chip was configured with a time interval of $\triangle t$ between computational channels, while the weight values were deployed on the OCU chip. Both the OMU and the OCU chips were optically packaged with polarization-maintaining single-mode fibers (PM SMFs) and connected via optical fiber connectors directly, eliminating the need for fiber polarization controllers (FPCs) for polarization management. Finally, the delayed and weighed signals were summed and amplified by a high-speed PD with TIA (Finisar XPRV2021). The output waveforms were recorded by a real-time oscilloscope (Tektronix DPO75902SX), and results were obtained by down sampling at a sampling interval of $4\triangle t$.

### Chip fabrication and packaging

The OCU chip was fabricated on the AMF’s multilayer Si_3_N_4_-on-SOI platform. The thicknesses of the BOX layer and the top Si layer are 3 μm and 220 nm, respectively, while both Si_3_N_4_ layers have a thickness of 400 nm. To minimize waveguide transmission loss, the Si₃N₄ waveguide layers were fabricated using a low-pressure chemical vapor deposition (LPCVD) process, and the measured insertion loss is less than 0.83 dB cm^-1^ for a single-mode waveguide with a 1-μm-width. The OMU chip was also fabricated by AMF based on a standard SOI platform, with a BOX thickness of 3 μm and a top silicon thickness of 220 nm. In the back end of the line (BEOL), both chips used two aluminum metal layers for electrical interconnection and wire bonding. A passivation layer with a thickness of about 4 μm was used to protect the chip surface from external contamination, moisture, and other environment factors, thereby enhancing device reliability. Both chips were packaged by the SJTU-Pinghu Institute of Intelligent Optoelectronics. All electrodes were wire bonded to printed circuit boards (PCBs) for electrical control. Fiber arrays were edge-coupled to the chips and fixed by ultraviolet-cured glue. To ensure thermal stability, a thermoelectric cooler (TEC) was also packaged beneath the chip, enabling temperature control with a resolution of 0.01 °C.

## Supplementary information

Supplementary for Integrated Photonic 3D Tensor Processing Engine

## Data Availability

The data that support the findings of this study are available from the corresponding author upon request.

## Code availability

The code used in the present work is available from the authors upon request.

## References

[1] LeCun, Y., Bengio, Y. & Hinton, G. Deep learning. *Nature* **521**, 436–444 (2015). 26017442 10.1038/nature14539

[2] Alzubaidi, L. et al. Review of deep learning: concepts, CNN architectures, challenges, applications, future directions. *Journal of Big Data* **8**, 53 (2021). 33816053 10.1186/s40537-021-00444-8 PMC8010506

[3] Ha, N. et al. Machine Learning-Enabled Smart Sensor Systems. *Advanced Intelligent Systems* **2**, 2000063 (2020).

[4] Ballard, Z. et al. Machine learning and computation-enabled intelligent sensor design. *Nature Machine Intelligence* **3**, 556–565 (2021).

[5] Sidiropoulos, N. D. et al. Tensor Decomposition for Signal Processing and Machine Learning. *IEEE Transactions on Signal Processing* **65**, 3551–3582 (2017).

[6] Li, Z. et al. A Survey of Convolutional Neural Networks: Analysis, Applications, and Prospects. *IEEE Transactions on Neural Networks and Learning Systems* **33**, 6999–7019 (2022). 34111009 10.1109/TNNLS.2021.3084827

[7] Kamnitsas, K. et al. Efficient multi-scale 3D CNN with fully connected CRF for accurate brain lesion segmentation. *Medical Image Analysis* **36**, 61–78 (2017). 27865153 10.1016/j.media.2016.10.004

[8] Niyas, S. et al. Medical image segmentation with 3D convolutional neural networks: A survey. *Neurocomputing* **493**, 397–413 (2022).

[9] Zhao, D. et al. An attentive and adaptive 3D CNN for automatic pulmonary nodule detection in CT image. *Expert Systems with Applications* **211**, 118672 (2023).

[10] Duan, H. et al. Revisiting Skeleton-based Action Recognition. *2022 IEEE/CVF Conference on Computer Vision and Pattern Recognition (CVPR*) (2022).

[11] De Castro, G. Z., Guerra, R. R. & Guimarães, F. G. Automatic translation of sign language with multi-stream 3D CNN and generation of artificial depth maps. *Expert Systems with Applications* **215**, 119394 (2023).

[12] Meng, Z. et al. HYDRO-3D: Hybrid Object Detection and Tracking for Cooperative Perception Using 3D LiDAR. *IEEE Transactions on Intelligent Vehicles* **8**, 4069–4080 (2023).

[13] Maturana, D. & Scherer, S. VoxNet: A 3D Convolutional Neural Network for real-time object recognition. *2015 IEEE/RSJ International Conference on Intelligent Robots and Systems (IROS)* (2015).

[14] Hong, Y.-Y. & Pula, R. A. Detection and classification of faults in photovoltaic arrays using a 3D convolutional neural network. *Energy* **246**, 123391 (2022).

[15] Faraji, M. An integrated 3D CNN-GRU deep learning method for short-term prediction of PM2.5 concentration in urban environment. *Sci. Total Environ.* (2022). 10.1016/j.scitotenv.2022.155324 35452742

[16] Li, Y., Zhang, H. & Shen, Q. Spectral–Spatial Classification of Hyperspectral Imagery with 3D Convolutional Neural Network. *Remote Sensing* **9**, 67 (2017).

[17] OpenAI. AI and compute. (2018). at https://openai.com/index/ai-and-compute/.

[18] OpenAI. AI and efficiency. at https://openai.com/index/ai-and-efficiency/ (2020).

[19] Mittal, S. & Vaishay, S. A survey of techniques for optimizing deep learning on GPUs. *Journal of Systems Architecture* **99**, 101635 (2019).

[20] Jouppi, N. P. et al. In-Datacenter Performance Analysis of a Tensor Processing Unit. *Proceedings of the 44th Annual International Symposium on Computer Architecture* (2017).

[21] Yao, P. et al. Fully hardware-implemented memristor convolutional neural network. *Nature* **577**, 641–646 (2020). 31996818 10.1038/s41586-020-1942-4

[22] Shawahna, A., Sait, S. M. & El-Maleh, A. FPGA-Based Accelerators of Deep Learning Networks for Learning and Classification: A Review. *IEEE Access* **7**, 7823–7859 (2019).

[23] Mittal, S. & Vibhu A survey of accelerator architectures for 3D convolution neural networks. *Journal of Systems Architecture* **115**, 102041 (2021).

[24] Miller, D. A. B. Attojoule Optoelectronics for Low-Energy Information Processing and Communications. *Journal of Lightwave Technology* **35**, 346–396 (2017).

[25] Boes, A. et al. Lithium niobate photonics: Unlocking the electromagnetic spectrum. *Science* **379**, eabj4396 (2023). 36603073 10.1126/science.abj4396

[26] Wan, Z. et al. Ultra-Degree-of-Freedom Structured Light for Ultracapacity Information Carriers. *ACS Photonics* **10**, 2149–2164 (2023).

[27] Shen, Y. et al. Deep learning with coherent nanophotonic circuits. *Nature Photonics* **11**, 441–446 (2017).

[28] Zhang, H. et al. An optical neural chip for implementing complex-valued neural network. *Nature Communications* **12**, 457 (2021). 10.1038/s41467-020-20719-7 PMC7815828 33469031

[29] Pai, S. et al. Experimentally realized in situ backpropagation for deep learning in photonic neural networks. *Science* **380**, 398–404 (2023). 37104594 10.1126/science.ade8450

[30] Zhan, Y. et al. Physics-Aware Analytic-Gradient Training of Photonic Neural Networks. *Laser & Photonics Reviews* **18**, 2300445 (2024).

[31] Feldmann, J. et al. Parallel convolutional processing using an integrated photonic tensor core. *Nature* **589**, 52–58 (2021). 33408373 10.1038/s41586-020-03070-1

[32] Huang, C. et al. A silicon photonic–electronic neural network for fibre nonlinearity compensation. *Nature Electronics* **4**, 837–844 (2021).

[33] Sludds, A. et al. Delocalized photonic deep learning on the internet’s edge. (2022). 10.1126/science.abq8271 36264813

[34] Zhang, W. et al. Broadband physical layer cognitive radio with an integrated photonic processor for blind source separation. *Nature Communications* **14**, 1107 (2023). 10.1038/s41467-023-36814-4 PMC9971366 36849533

[35] Dong, B. et al. Higher-dimensional processing using a photonic tensor core with continuous-time data. *Nature Photonics* **17**, 1080–1088 (2023).

[36] Dong, B. et al. Partial coherence enhances parallelized photonic computing. *Nature* **632**, 55–62 (2024). 39085539 10.1038/s41586-024-07590-y PMC11291273

[37] Xu, X. et al. 11 TOPS photonic convolutional accelerator for optical neural networks. *Nature* **589**, 44–51 (2021). 33408378 10.1038/s41586-020-03063-0

[38] Xu, S. et al. High-order tensor flow processing using integrated photonic circuits. *Nature Communications* **13**, 7970 (2022). 10.1038/s41467-022-35723-2 PMC9797566 36577748

[39] Bai, B. et al. Microcomb-based integrated photonic processing unit. *Nature Communications* **14**, 66 (2023). 10.1038/s41467-022-35506-9 PMC9814295 36604409

[40] Zhang, J. et al. Compact, efficient, and scalable nanobeam core for photonic matrix vector multiplication. *Optica*, 10.1364/OPTICA.506603 (2024).

[41] Li, X. et al. Ultra-low-loss multi-layer 8 × 8 microring optical switch. *Photonics Research* **11**, 712 (2023).

[42] Li, X. et al. Low-Loss and Power-Efficient Polarization-Diversity 4 × 4 Microring Switch on a Multi-Layer Si3N4-on-SOI Platform. *J. Lightwave Technol*. 1–10, (2024).

[43] Zhang, W. et al. Silicon microring synapses enable photonic deep learning beyond 9-bit precision. *Optica* **9**, 579 (2022).

[44] Liu, X. et al. Single-Monitor Calibration for Multiple Microring Synapses. *ACS Photonics*, acsphotonics.4c00157, 10.1021/acsphotonics.4c00157 (2024).

[45] Cheng, J. et al. Self-calibrating microring synapse with dual-wavelength synchronization. *Photonics Research* **11**, 347 (2023).

[46] Che, D. & Chen, X. Modulation format and digital signal processing for IM-DD optics at post-200G era. *Journal of Lightwave Technology* **42**, 588–605 (2024).

[47] Guo, Y. et al. Deep Learning for 3D Point Clouds: A Survey. *IEEE Transactions on Pattern Analysis and Machine Intelligence* **43**, 4338–4364 (2021). 32750799 10.1109/TPAMI.2020.3005434

[48] Deuge, M. D. et al. Unsupervised feature learning for classification of outdoor 3D scans. *Australasian conference on robitics and automation.* **2**, 1 (2013).

[49] De Koninck, Y. et al. GaAs nano-ridge laser diodes fully fabricated in a 300-mm CMOS pilot line. *Nature* **637**, 63–69 (2025). 39743604 10.1038/s41586-024-08364-2

[50] Shu, H. et al. Microcomb-driven silicon photonic systems. *Nature* **605**, 457–463 (2022). 35585341 10.1038/s41586-022-04579-3 PMC9117125

[51] Sobhanan, A. et al. Semiconductor optical amplifiers: recent advances and applications. *Advances in Optics and Photonics* **14**, 571 (2022).

[52] Yang, X. et al. Non-Volatile Optical Switch Element Enabled by Low-Loss Phase Change Material. *Adv. Funct. Materi.* 2304601, (2023).

[53] Hong, S. et al. Multimode-enabled silicon photonic delay lines: Break the delay-density limit. *Light: Science & Applications* **14**, 145 (2025). 10.1038/s41377-025-01820-2 PMC11958738 40164583

