# Supplementary material for: Integrated photonic 3D tensor processing engine
Source: Light Sci Appl. 2026 Mar 6;15:154. doi: 10.1038/s41377-026-02183-y (PMC12966298; doi:10.1038/s41377-026-02183-y)
Supplement: Supplementary file 1 — Supplementary for Integrated Photonic 3D Tensor Processing Engine [file 41377_2026_2183_MOESM1_ESM.docx]

**Supplementary information for integrated photonic 3D tensor processing engine**

Yue Wu^1,†^, Ziheng Ni^1,†^, Xin Li^1^, Yuanxun Wang^1^, Liangjun Lu^1,2^, Jianping Chen^1,2^, and Linjie Zhou^1,2^

^1^State Key Laboratory of Photonics and Communications, School of Integrated Circuits, Shanghai Jiao Tong University, Shanghai 200240, China,

^2^SJTU-Pinghu Institute of Intelligent Optoelectronics, Pinghu 314200, China,

^†^These authors contributed equally: Yue Wu, Ziheng Ni

[luliangjun@sjtu.edu.cn](mailto:luliangjun@sjtu.edu.cn); [ljzhou@sjtu.edu.cn](mailto:ljzhou@sjtu.edu.cn)

[Supplementary note 1: 3D tensor processing engine across resonance orders 2](#_Toc192151544)

[Supplementary note 2: Dual-coupled-MRRs weighting element 4](#_Toc192151545)

[Supplementary note 3: MNIST recognition task 7](#_Toc192151546)

[Supplementary note 4: Design and test of the optical memory unit chip 9](#_Toc192151547)

[Supplementary note 5: Signal synchronization between multi-channels 11](#_Toc192151548)

[Supplementary note 6: Real-time waveform comparison 12](#_Toc192151549)

[Supplementary note 7: Computing energy efficiency estimation 13](#_Toc192151550)

[Supplementary note 8: Insertion loss of the 3D CNN 16](#_Toc192151551)

[Supplementary note 9: Compute density evaluation 17](#_Toc192151552)

[Supplementary note 10: Scalability of the system 17](#_Toc192151553)

[Reference 21](#_Toc192151554)

# Supplementary note 1: 3D tensor processing engine across resonance orders

In the main text, in order to avoid power perturbation induced by coherent interference between paths at identical wavelengths, photodetectors are used at the output ports of the optical tunable delay lines to convert optical signals to electrical signals, and signal accumulation is performed in the electrical domain with an electrical power combiner. Alternatively, signal accumulation across optical paths can occur directly in the optical domain by leveraging the rich resonance orders of the MRRs.

Fig. S1a illustrates the working principle of the proposed all-optical 3D tensor processing engine (3D-TPE). To perform tensor convolution between two 3D matrices ***A*** and ***B***, each with dimensions ($I,J,K)$ where $I\times J\geq K$, the input matrix ***A*** is sequentially encoded onto multi-wavelength carriers by a high-speed modulator with a symbol duration of $\Delta t$. The number of wavelengths is $I\times J\times K$, corresponding to the $K$ resonance orders of the MRR (from *m*^th^ to (*m*+*K*-1)^th^ orders), where each resonance order contains $I\times J$ operating wavelengths, as shown in Fig. S1b. The modulated optical carriers are then divided into $K$ paths using a WDM with channel spacing equal to the free spectral range (FSR) of the MRR. After passing through $K$ tunable delay lines with equal time intervals of $\Delta t$, the replicas enter the dual-coupled-MRRs crossbar optical computing unit (OCU). The OCU comprises $K$ rows and $I\times J$ columns, with operating wavelengths configured such that different rows function at distinct resonance orders, while different columns in each row exhibit varying resonance wavelengths of the same order, as illustrated in Fig. S1c. The weights of matrix ***B*** are deployed on the dual-coupled-MRRs crossbar circuit. Utilizing the filtering effect of MRRs, weighted replicas from different rows, with time intervals of $\Delta t$, combine into vertical bus waveguides and are transmitted into another array of optical tunable delay lines with time intervals of $K\Delta t$. After the second time delay, all signals are combined via an optical combiner. Consequently, the $I\times J\times K$ weighted replicas across multiple wavelengths have uniform delay time intervals of $\Delta t$ after traversing the $I\times J\times K$ weighting channels. Finally, a high-speed photodetector (PD) performed the summation. The 3D convolutional processing results are derived by sampling the PD output waveforms at a rate of $1/(I\times J\times K)\Delta t$. As the $I\times J\times K$ delayed signals operate at distinct wavelengths, they can be directly combined in the optical domain without concern for phase perturbation among different paths of identical wavelengths.

In contrast with main text scheme, the supplementary architecture enables fully all-optical computation, potentially offering higher system bandwidth by avoiding the limitations associated with electrical summation. However, it requires *N×N* laser wavelengths and an *N×*1 optical combiner, which introduces higher insertion loss and increased power overhead due to the additional laser resources. Despite these concerns, the supplementary architecture holds promise for future implementations with the advancements of laser and optical frequency comb technologies.


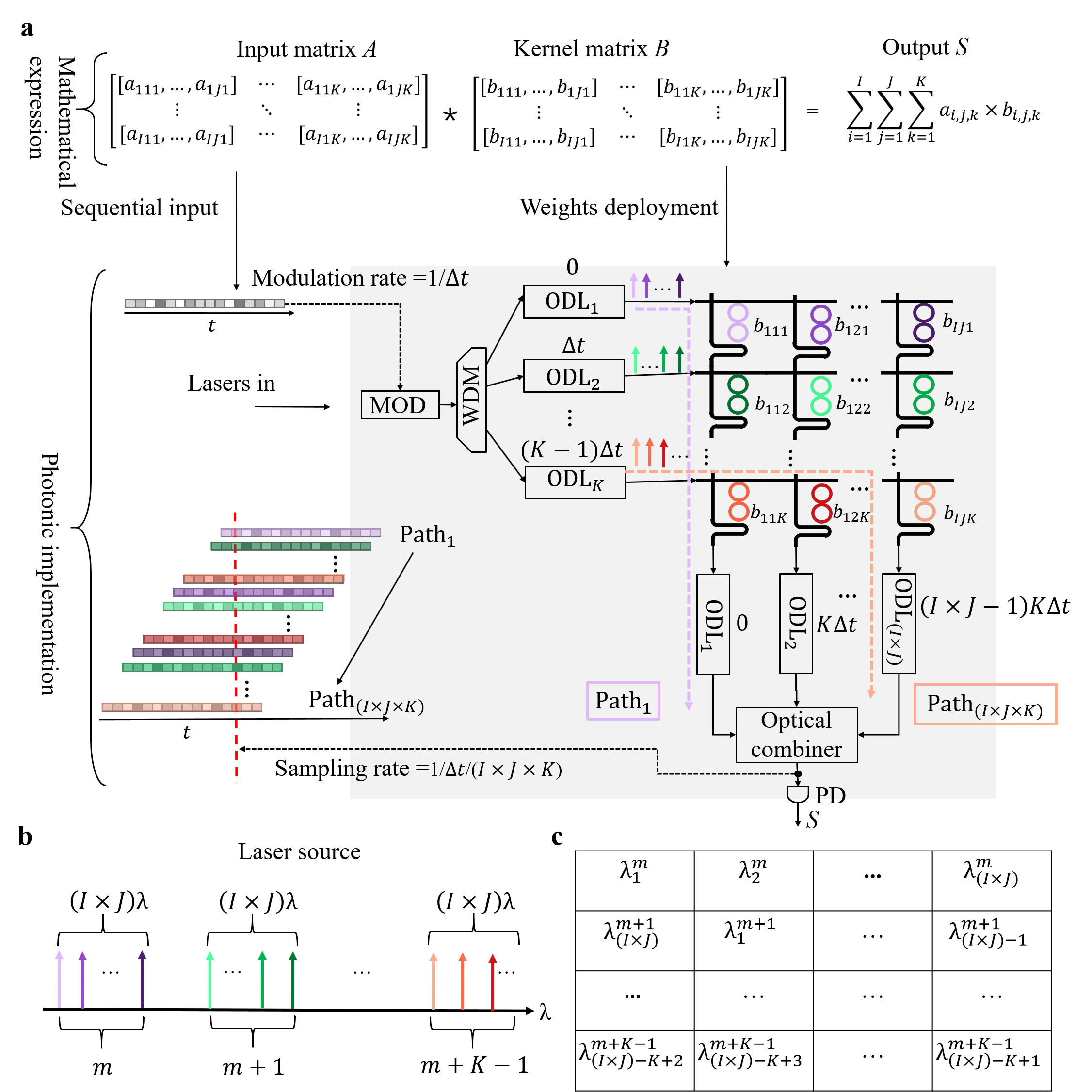


**Fig. S1 Processing flow of the all-optical 3D-TPE**. **a.** The 3D matrix ***A***, with a size of ($I,J,K)$, is encoded onto the amplitude of multi-wavelength carries by sequential modulation. The 3D kernel matrix ***B***, with a size of ($I,J,K)$, is deployed on the dual-coupled-MRRs based crossbar circuit. The 3D tensor convolution operation is accomplished by controlling time delay intervals between channels and the coding process. **b.** The multi-wavelength laser source consists of $I\times J\times K$ wavelengths corresponding to the $K$ resonance orders of the MRR, with each order containing $I\times J$ operating wavelengths. **c.** Operating wavelength configuration of the crossbar OCU chip.

# Supplementary note 2: Dual-coupled-MRRs weighting element

Fig. S2 shows the schematic structure and the cross sections of the proposed dual-coupled-MRRs weighting element (WE). The dual-coupled MRRs were fabricated on a multilayer ${Si}_{3}N_{4}$-on-SOI platform, with two MRRs located in the middle ${Si}_{3}N_{4}$ layer. The MRR incorporates a 1-μm-long straight waveguide in each coupling region. In order to reduce bend-to-straight transition loss in the ring, four 90° bends are designed as Euler bends, thus decreasing insertion loss at the drop port. The FSR of the MRRs is 4.8 nm.


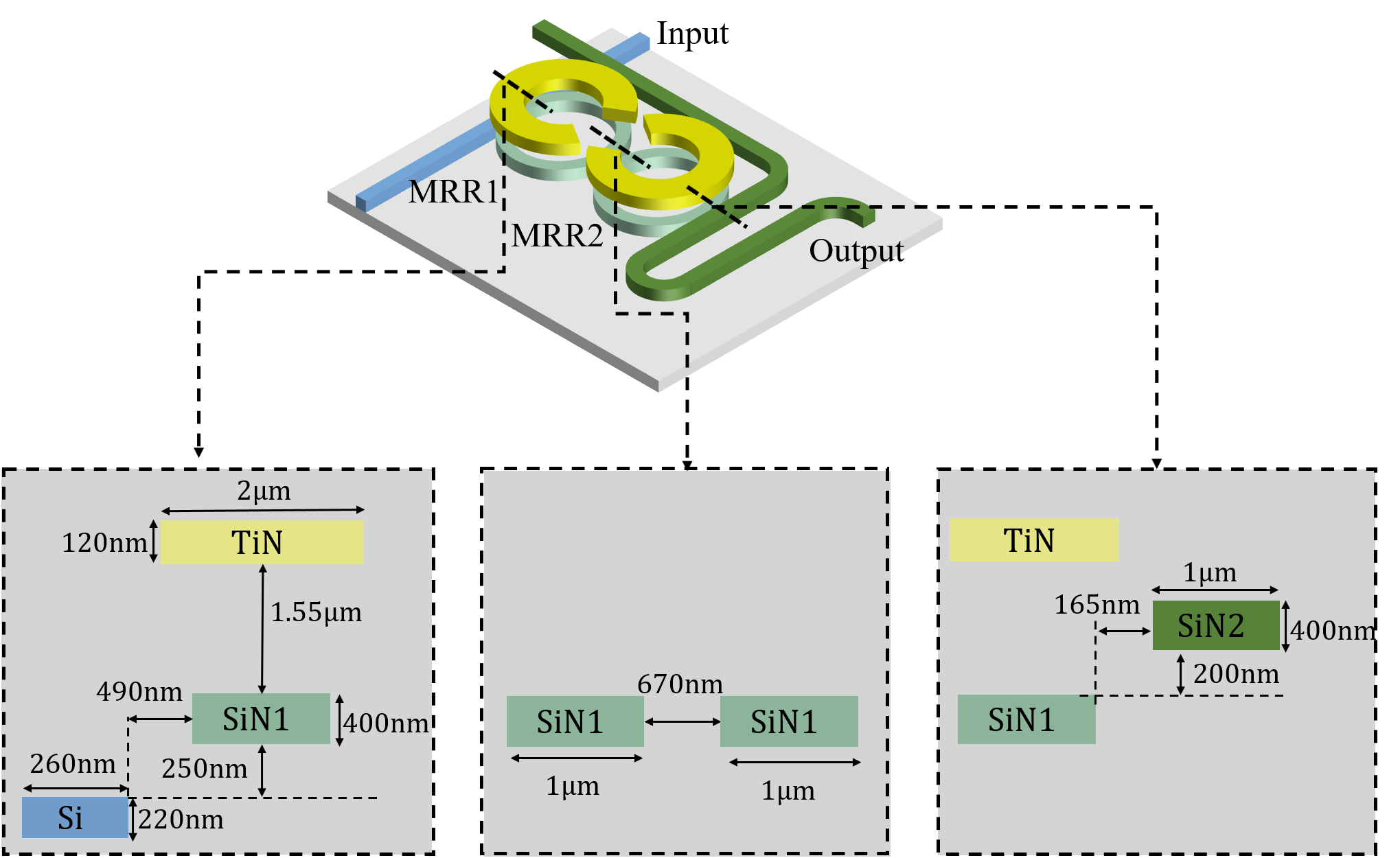


**Fig. S2** Structure of the dual-coupled-MRRs WE and cross-sectional views of the coupling regions with key dimensions indicated.


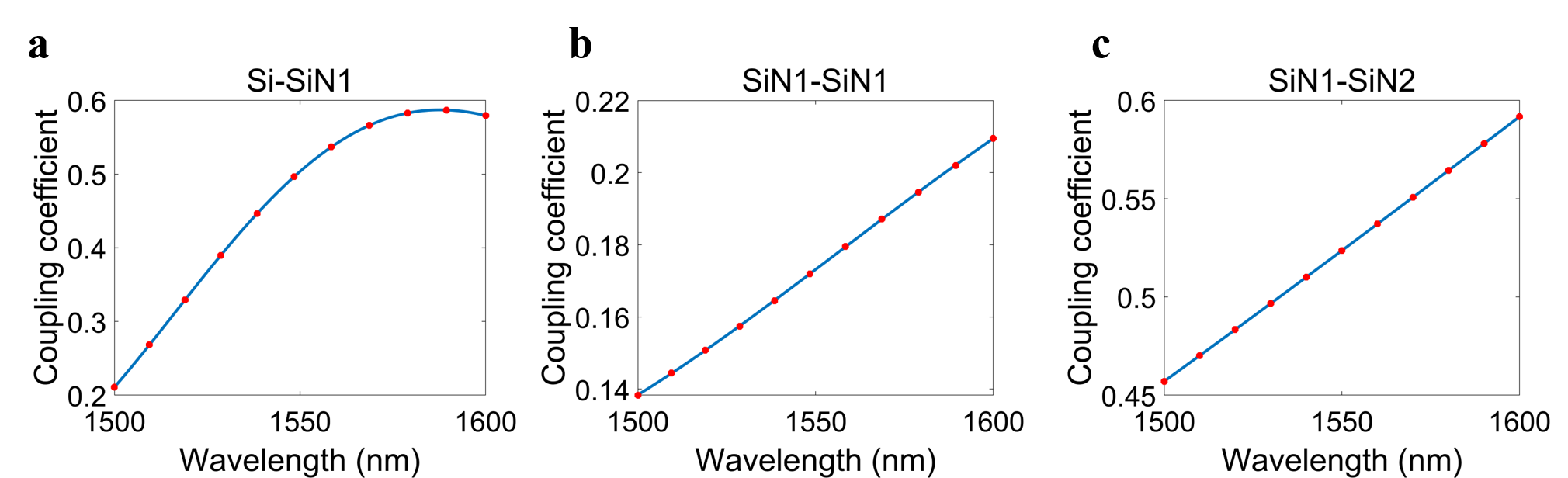


**Fig. S3** **Simulated coupling coefficients of the three coupling regions in the dual-coupled-MRRs WE. a.** Coupling between the Si bus waveguide and the middle ${Si}_{3}N_{4}$ MRR (marked as SiN1). **b.** Coupling between two MRRs in the middle ${Si}_{3}N_{4}$ layer. **c.** Coupling between the middle ${Si}_{3}N_{4}$ MRR and the top ${Si}_{3}N_{4}$ bus waveguide (marked as SiN2).

The simulation of the dual-coupled-MRRs WE focuses on three coupling regions, including the coupling region between the Si bus waveguide and the middle ${Si}_{3}N_{4}$ MRR, the coupling region between the two MRRs, and the coupling region between the middle ${Si}_{3}N_{4}$ MRR and the top ${Si}_{3}N_{4}$ bus waveguide. Fig. S3 shows the simulated coupling coefficient of the three coupling regions using the 3D FDTD method.


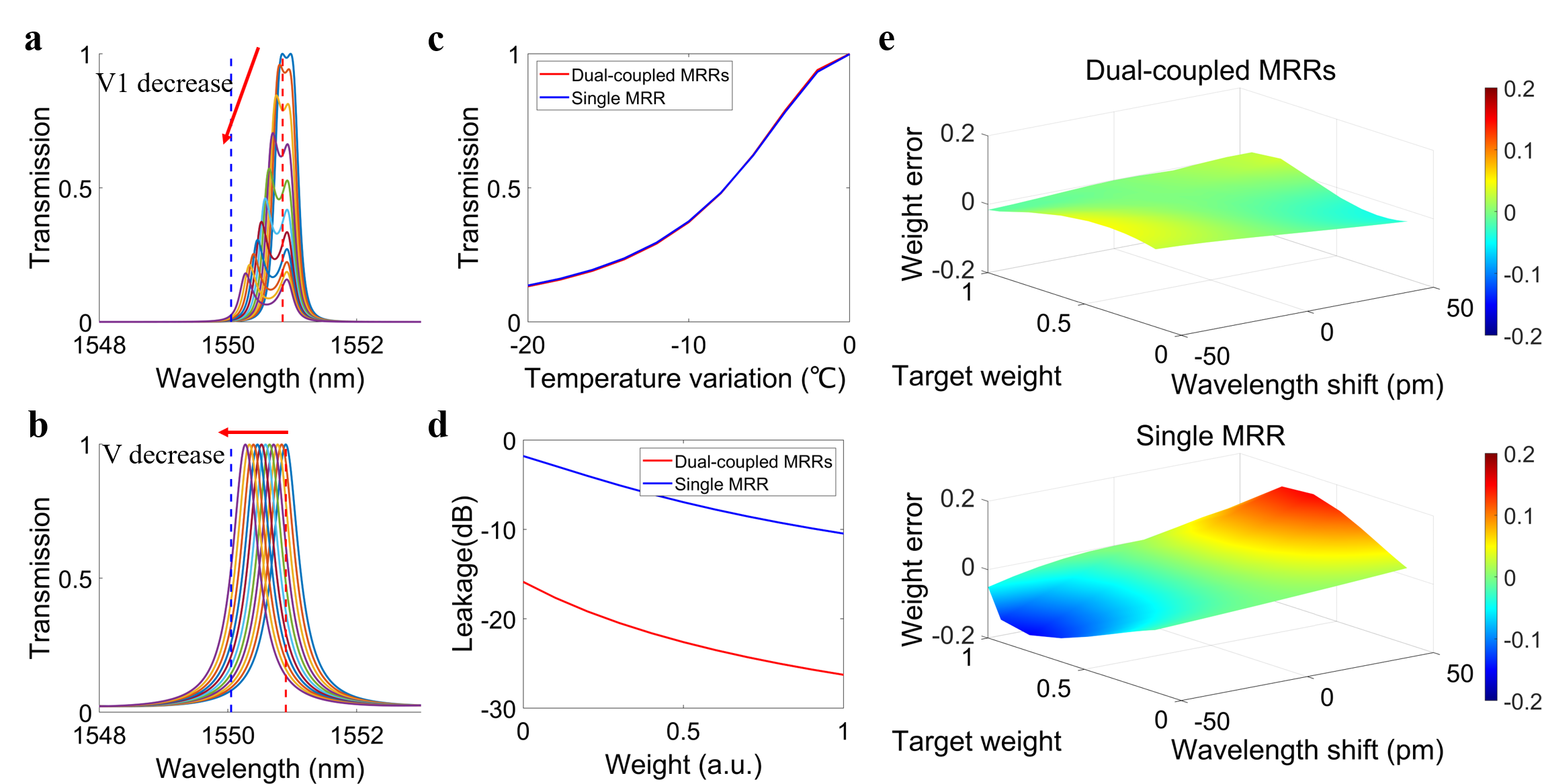


**Fig. S4** **Comparison between the dual-coupled-MRRs WE and a single-MRR WE.** **a.** Transmission spectra of the dual-coupled-MRRs WE were obtained by sweeping the voltages of MRR1. **b.** Transmission spectra of the single MRR. The red (blue) dotted lines in **a** and **b** illustrate the operating (adjacent) wavelengths for weighting. **c.** Weight tuning under different temperature variations for the dual-coupled MRR WE and the single MRR WE, respectively. The temperature variation at a weight value of 1 is marked as 0. **d.** Optical leakage from adjacent wavelength channels during the weight-tuning process. **e.** Weight errors upon the laser wavelength drift for the dual-coupled-MRRs WE (upper panel) and the single-MRR WE (bottom panel), respectively.

The transmission spectrum of the dual-coupled-MRRs WE was simulated using the transfer matrix method by importing the simulated coupling coefficients into the matrix. The spectrum shows a flat-top characteristic with a 3 dB bandwidth exceeding 50 GHz. Suppl. Figs. 4a and 4b illustrate the weight-tuning process of the dual-coupled-MRRs WE and a single MRR. Voltage variation is emulated by adjusting the temperature. As the temperature changes, the refractive index of the waveguide is modified, thereby altering the transmission spectral characteristics. The thermo-optic coefficient of the ${Si}_{3}N_{4}$ waveguide used here is 4.098×10^-5^. Fig. S4c illustrates the relationship between optical transmission and temperature variations for both a dual-coupled-MRRs WE and a single-MRR WE, both exhibiting similar weight tuning efficiency. By normalizing the maximum and minimum output optical power across all WEs, weight values between 0 and 1 can be achieved. The normalization formula is expressed as follows:

$$w=\frac{T-T_{min}}{T_{max}-T_{min}}$$

where $w$ is the weight value, $T$ is the optical power at the operating wavelengths, $T_{max}$ and $T_{min}$ are the maximum and minimum output optical power across all WEs within voltage sweeping range, respectively. Real-number computation can be further implemented by incorporating reference paths^1^, balanced photodetectors^2^, or decomposing a real number into two positive values^3^.

Due to the limited roll-off ratio of MRR-based WEs, optical leakage from adjacent wavelength channels will lead to unexpected optical power accumulation, thus degrading computing accuracy. Optical power leakage from an adjacent wavelength channel (indicated by the blue dotted lines in Suppl Figs. 4a and 4b) with a channel spacing of 100 GHz was observed while tuning the weights of the operating wavelength channel (as indicated by the red dashed lines in Suppl Figs. 4a and 4b), as shown in Suppl Fig. 4d. Weight tuning of the MRR-based WE results in the variations of optical leakage, which increases with the decrease of weight values. Nonetheless, optical leakage from adjacent channels in the dual-coupled-MRRs WE is significantly lower than in the single-MRR WE due to the faster roll-off, indicating smaller weight errors.

Given that laser emission wavelength drifts may occur due to temperature and pump current fluctuations, we monitored the output optical power under operating wavelength drifts ranging from ±50 pm and calculated the deviations from ideal optical power as weight errors. Suppl Fig. 4e shows the relationship between weight error and wavelength drift. For a single-MRR WE, the weight error increases rapidly with wavelength drift, with a 50 pm drift resulting in a maximum weight error of 0.152. In comparison, the impact of wavelength drift on weight error is significantly mitigated in the dual-coupled-MRRs WE, exhibiting smoother weighting characteristics and a maximum weight error of 0.067 for a 50 pm wavelength drift. The dual-coupled-MRRs WE shows larger weight errors at lower weight values due to the flatter spectral characteristics at higher weight values. Utilizing the weight pruning technique^4^, which emphasizes higher weight values in neural network kernel weights, can minimize the impact of errors at smaller weight values.

# Supplementary note 3: MNIST recognition task

To verify the parallel computational capability of the dual-coupled-MRRs based optical computing unit (OCU) chip, a 10-category handwritten digit recognition task based on the MINIST dataset was performed. The convolutional neural network (CNN) architecture is shown in Fig. S5. The convolution layer consists of four 2×2 convolution kernels constrained to positive values for extracting image features. The MVM operation within the convolution layer was executed optically on the OCU chip, while other operations were accomplished by a digital computer. Fig. S6 illustrates the experimental setup. In the experiment, four modulators (ixblue MXAN-LN-20) were used to modulate four weighting channels in parallel. Synchronization between channels was done by the internal clock module of a high-speed arbitrary waveform generator (AWG, Keysight M8199A). After the training process, the trained kernel weights were deployed on the OCU chip according to the W-V relationships via a look-up-table method. The weight voltages were provided by a multi-channel voltage source. During image inference, images were initially patched according to the kernel dimensions and then modulated into the optical domain by the modulators and the AWG. MVM operation was completed as signals passed through the OCU chip. The accumulated optical signals were then converted into electrical signals by a high-speed photodetector (PD) equipped with a trans-impedance amplifier (TIA) (Finisar XPRV2021) and recorded by a real-time oscillograph (OSC) (Tektronix DPO75902SX). Fig. S7 compares some randomly selected feature maps obtained from the optical implementation and a digital computer, revealing slight differences. These results demonstrate the promising potential of our OCU chip in parallel computing and image recognition applications.


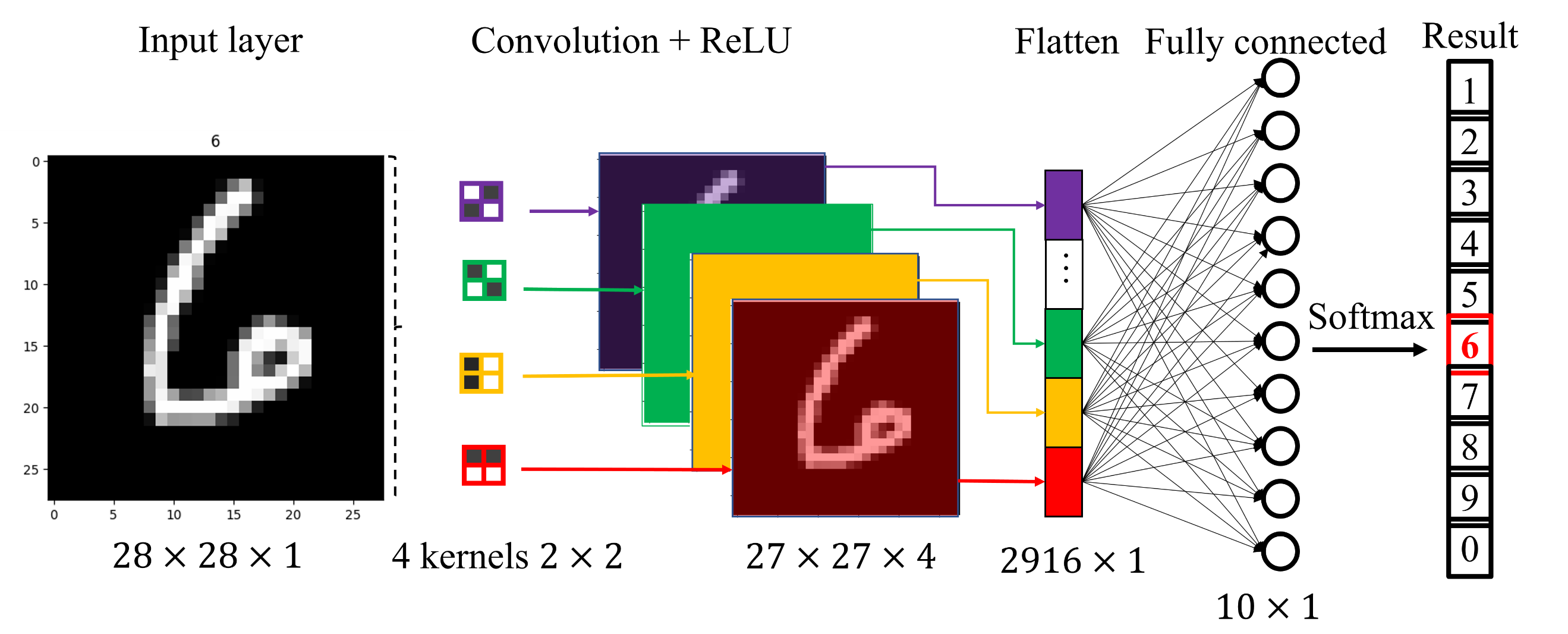


**Fig. S5** CNN architecture for 10-category MNIST handwritten digit recognition task.


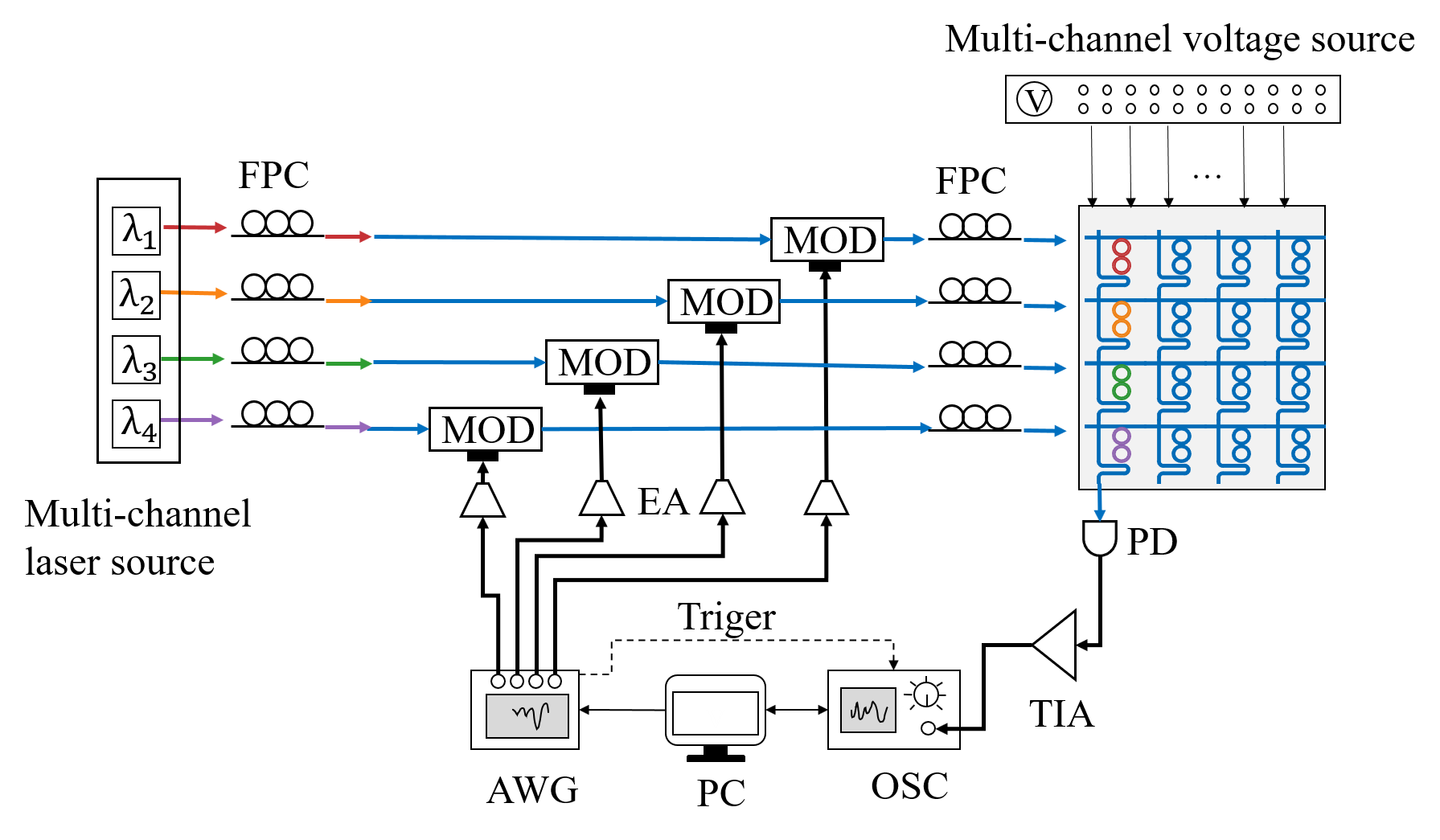


**Fig. S6** **Experimental setup for 10-category MNIST handwritten digit recognition task.** FPC: fiber polarization controller; MOD: modulator; AWG: arbitrary waveform generator; EA: electrical amplifier; PC: personal computer; OSC: oscilloscope; TIA: trans-impedance amplifier; PD: photodetector.


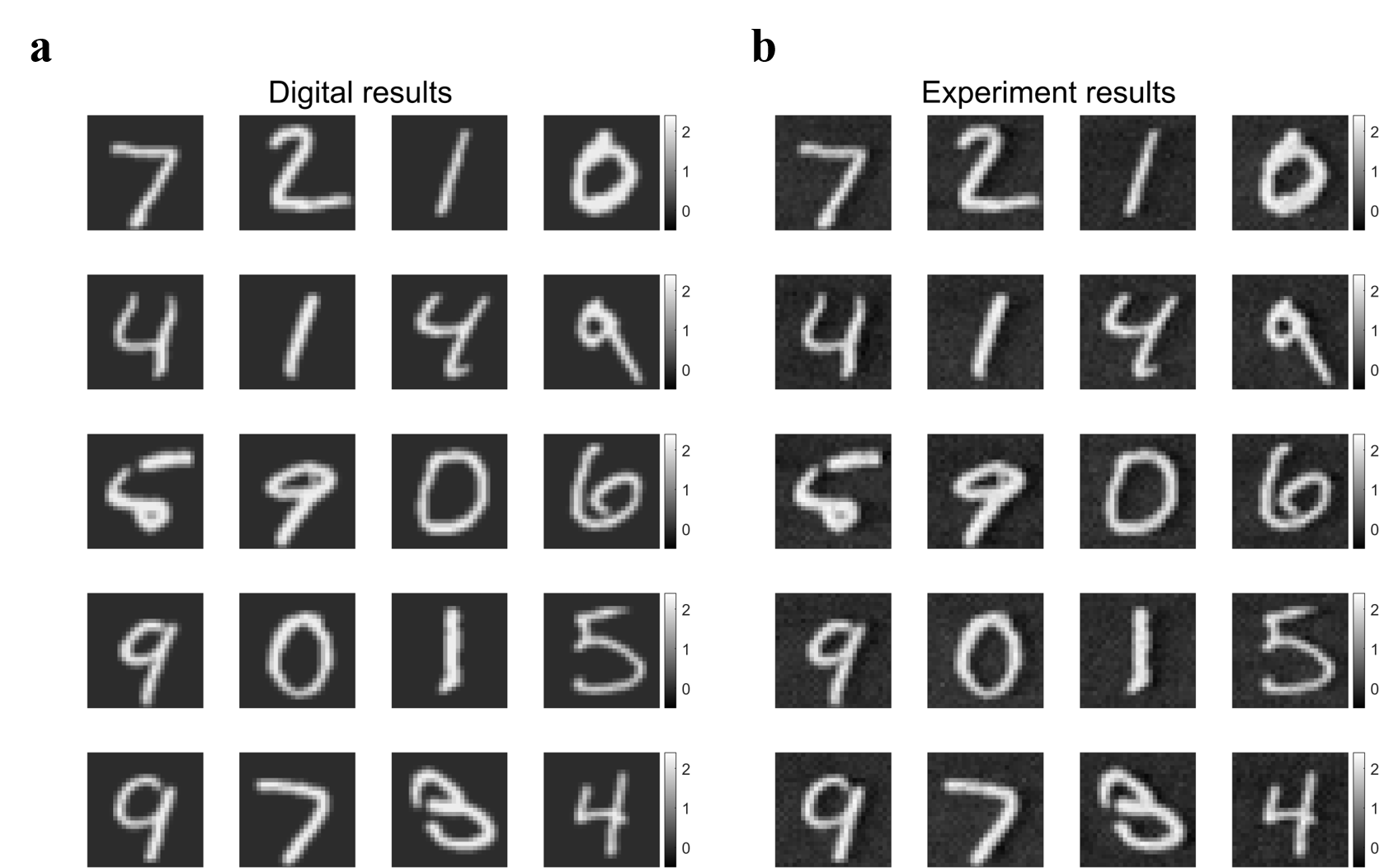


**Fig. S7** Comparison of the convolutional feature maps calculated by **a.** the digital computer and **b.** the optical computing chip.

# Supplementary note 4: Design and test of the optical memory unit chip

The optical memory unit (OMU) chip consists of eight identical tunable optical true-time delay lines (OTTDLs). Fig. S8a displays the schematic of the 6-bit switchable tunable delay line, which includes 7 MZI-based optical switches to digitally select delay paths with varied lengths to realize discrete delay tuning. The delay waveguide width is tapered from 0.5 μm to 2 μm to reduce transmission loss and delay errors induced by fabrication deviation. Fig. S8b illustrates the cross-section of the 2-μm-wide ridge waveguide. Each optical switch consists of two 2×2 multimode interferometers (MMIs) and two waveguide arms. A TiN microheater is integrated on one of the arms for thermo-optic (TO) phase shift. Furthermore, we insert two variable optical attenuators (VOAs) based on PIN diodes following each pair of delay waveguides for delay state calibration^5^. Fig. S8c illustrates the cross-section of the VOA.

**
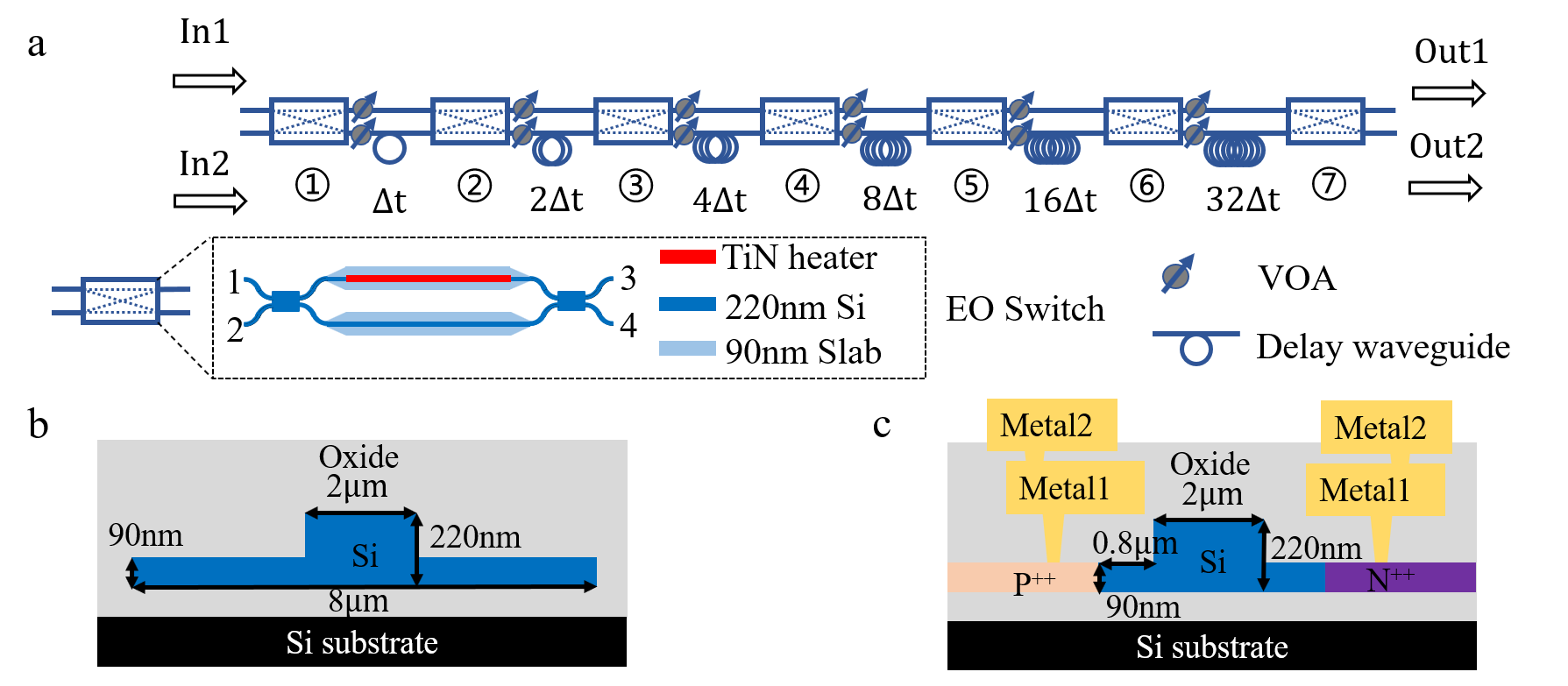
**

**Fig. S8** **Design of the OTTDL.** **a.** Schematic of the OTTDL. **b.** Cross sections of the 2-μm-wide ridge waveguide. **c.** PIN-diode-based VOA.

Fig. S9a depicts the experimental setup for group delay measurement. A continuous wave (CW) laser (Agilent 8164A) was modulated by an electro-optic intensity modulator (iXblue MXAN-LN-20) biased at the quadrature point. The driving RF signals were generated from a vector network analyzer (VNA, Anritsu MS46522B). The modulated optical signal was then coupled into our delay line chip and subsequently amplified by an erbium-doped fiber amplifier (EDFA). The delayed optical signal was finally converted to an electrical signal by a photodetector (PD, Finisar XPDV3120R). The group delay responses were acquired by the VNA.

Fig. S9b presents the measured group delay responses of all 64 delay states of the first channel OTTDL. Fig. S9c shows the delay error statistics of all the 64 delay states across the 8-channel OTTDLs at the 43.5 GHz frequency. The delay error is defined as the difference between the measured and the target group delays. We achieved a delay error of less than 0.6 ps for all the states^5^. The high delay accuracy of our OMU chip guarantees the computing accuracy of our 3D-TPE.


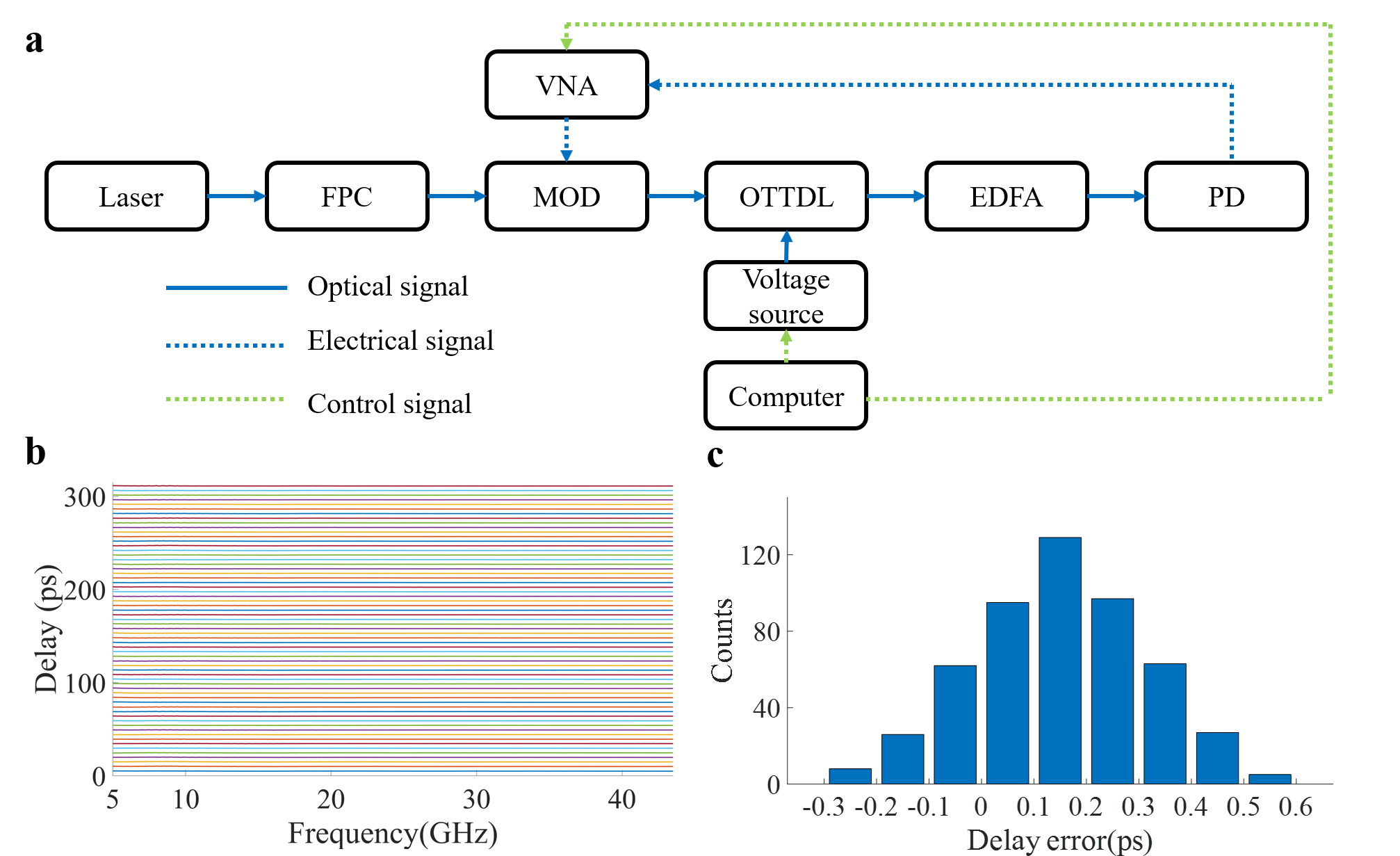


**Fig. S9 Group delay performance of the OMU chip. a.** Experimental setup of the group delay measurement. **b.** Group delay responses of all the 64 delay states of the first OTTDL. **c.** Delay error statistics of all the delay states of the chip. FPC: fiber polarization controller; EDFA: erbium-doped fiber amplifier; PD: photodetector; VNA: vector network analyzer.

# Supplementary note 5: Signal synchronization between multi-channels

Synchronous calibration across weighting channels is necessary before performing optical computing. Since the transmitted waveforms are identical for all channels, we only need to tune the relative delay between output waveforms of different channels without tuning the weights. A step signal was used to monitor the timestamp alignment between channels. The delay lines in the OMU chip were configured to the shortest paths, and the dual-coupled-MRRs WEs on the OCU chip were set to a weight value of 1 to ensure maximum output optical power. By controlling the laser source on or off in each corresponding channel, we recorded the output waveforms for each of the four channels individually, as shown in Fig. S10a. Initial delay differences are randomly distributed due to the length differences of connecting fibers between the weighting channels. After the reconfiguration of the MZIs’ switching states in the delay lines to select proper optical paths, a uniform delay interval across four channels is obtained, as shown in Fig. S10.


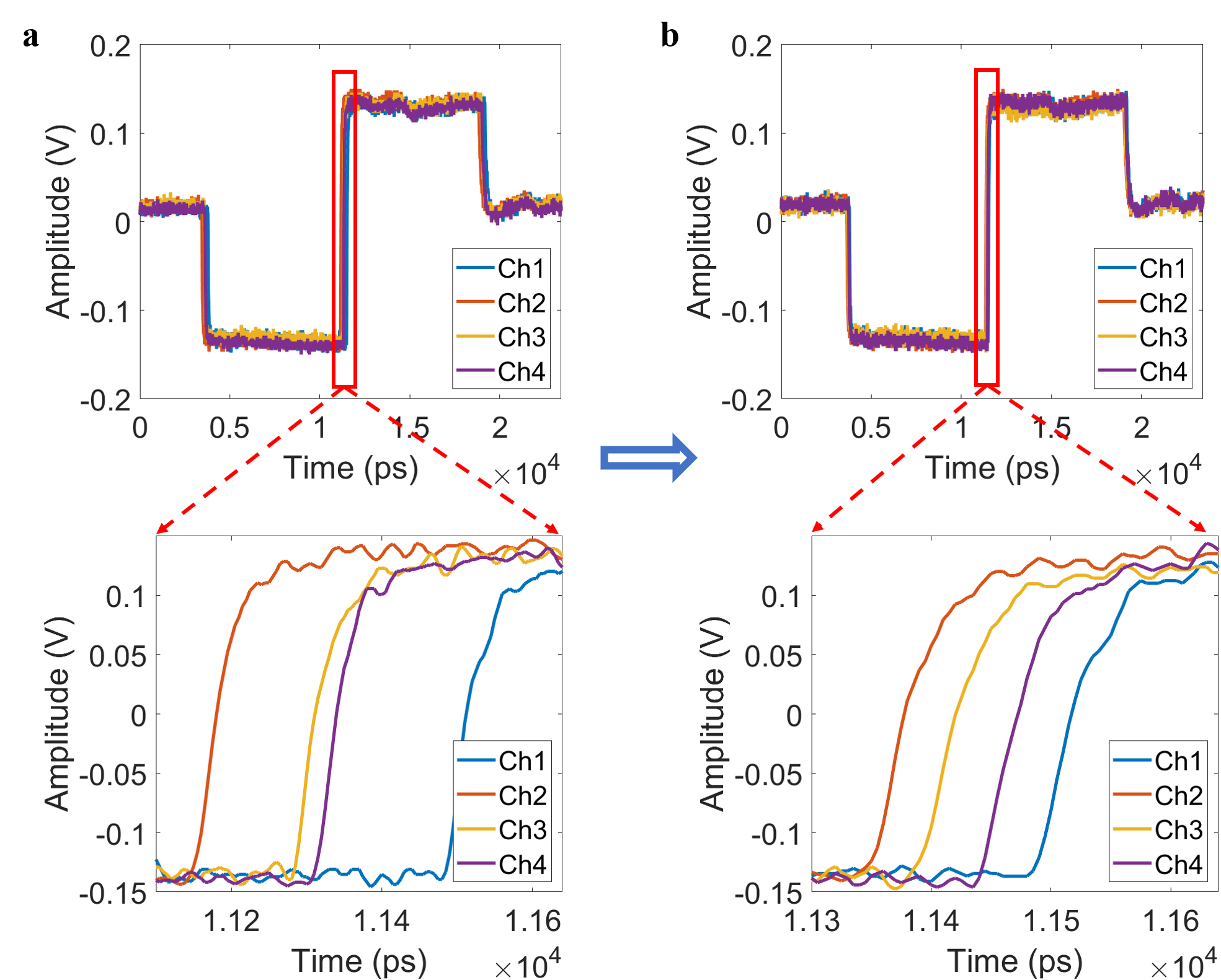


**Fig. S10** **Weighting channel synchronization calibration.** **a.** Before calibration, the delay intervals between different channels are randomly distributed. **b**. After calibration, there is a uniform time interval between channels.

# Supplementary note 6: Real-time waveform comparison

More comparison between the experiment waveforms and the calculated results are shown below.


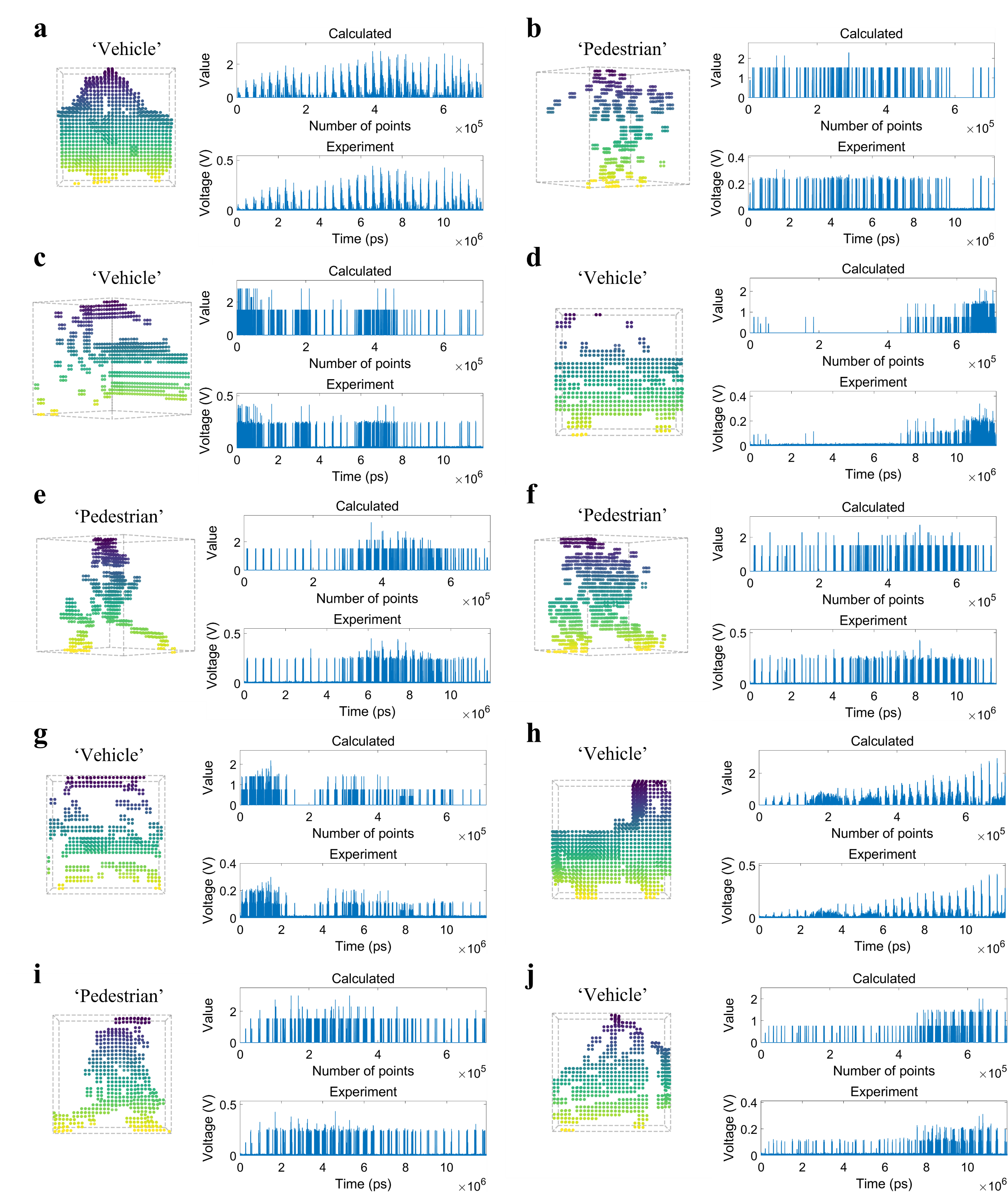


**Fig. S11 a-h** Visualization feature map of vehicle or pedestrian class (left panel), and comparison between the corresponding digitally calculated waveform (top right panel) and the experimental waveform (bottom right panel).

# Supplementary note 7: Computing energy efficiency estimation

In the 3D-TPE, power consumption mainly comes from the following parts: (ⅰ) ADC and DAC; (ⅱ) modulator and photodetector; (ⅲ) dual-coupled-MRRs based OCU chip; (ⅳ) tunable optical delay line-based OMU chip; (v) laser source.

Benefiting from the rapid development of advanced CMOS processes, high-performance transceivers with integrated high-speed ADCs and DACs have reached bit rates exceeding 200 Gb s^-1^, supporting high-speed analog-to-digital and digital-to-analog conversion. Table. S1 summarizes the performance of recent high-performance transceivers. For data rates above 200 Gb s^-1^, a DAC-based transmitter (TX) has an average power efficiency of 1 pJ b^-1^ at PAM4 modulation, equating to 2 pJ per conversion, while an ADC-based receiver (RX) has a power efficiency of approximately 2.8 pJ per conversion (1.4 pJ b^-1^).

Table. S1. State-of-the-art high-performance transceivers

| Year | Process | Data rate (Gb s^-1^) | Modulation format | TX APE (pJ b^-1^) | RX APE (pJ b^-1^) | Resolution  (bits) |
| --- | --- | --- | --- | --- | --- | --- |
| 2024^6^ | 3nm | 224 | PAM4 | 0.92 | / | 7 |
| 2024^7^ | 3nm | 224 | PAM4 | 1 | 2 | 7 |
| 2024^8^ | 5nm | 212 | PAM4 | 1.33 | 1.36 | 7 |
| 2024^9^ | 5nm | 200 | / | / | 2 | 8 |
| 2022^10^ | 5nm | 224 | PAM4 | / | 1.41 | 6 |
| 2021^11^ | 10nm | 224 | PAM4 | 1.88 | / | 7 |
| 2021^12^ | 7nm | 112 | PAM4 | 1.4pJ | / | 8 |
| 2021^13^ | 7nm | 112 | PAM4 | / | 3.18 | 7 |

^APE: analog power efficiency^

For high-speed modulators and photodetectors, energy consumption mainly comes from high-speed linear drivers and TIAs, which are approximately 1.08 pJ Sa^-1^ (224 Gb s^-1^ PAM4)^14^ and 0.84 pJ Sa^-1^ (112 Gb s^-1^ PAM4)^15^, respectively.

Power consumption for the OCU chip and the OMU chips is primarily dedicated to state maintenance. The average power consumption of a dual-coupled-MRRs WE is approximately 75.9 mW. For the optical tunable delay line, the power consumption of thermal phase adjustment is approximately 10 mW. For the 6-bit delay adjustment, a total of 7 switches are used, corresponding to a maximum power consumption of 10 mW×7=70 mW.

The output power of a single-wavelength laser is considered to be ~12 dBm (15.8 mW). The power consumption of the single-wavelength laser is about ~318 mW if a 5% wall-plug efficiency is considered.

In summary, for a 3D-TPE at a size of $N_{r}\times N_{r}$, operating at a clock frequency of $f_{s}$ (GHz), the expected power consumption $P$ and computing speed *R* are given by the following equations:

$$P(\mathrm{mW})= \left( 2+2.8+1.08+0.84 \right)\times f_{s}+75.9\times{N_{r}}^{2}+70\times2\times N_{r}+\left( 318 \right)\times N_{r}$$

$$R(\mathrm{GHz})={2\times N_{r}}^{2}\times f_{s}$$

The power efficiency can be expressed as:

$$\frac{R}{P}=\frac{{2\times N_{r}}^{2}\times f_{s}}{6.72{\times f}_{s}+75.9{\times N_{r}}^{2}+458\times N_{r}}$$

In our experiments, $f_{s}$= 30 GHz, $N_{r}=4$, the energy efficiency is calculated as 0.96 TOPS/3.248 W=0.30 TOPS W^-1^. For $f_{s}$= 200 GHz, $N_{r}=9$, the energy efficiency is calculated as 32.4 TOPS/11.614 W=2.79 TOPS W^-1^.

Combined with non-volatile phase-change materials (PCM)^16^, zero static power consumption can be achieved to maintain phase states. Power consumption for the dual-coupled-MRRs based OCU chip and the optical tunable delay line-based OMU chip can be neglected, and the power efficiency can be further expressed as:

$$\frac{R}{P}=\frac{{2\times N_{r}}^{2}\times f_{s}}{6.72\times f_{s}+318\times N}$$

For $f_{s}$= 30 GHz, $N_{r}=4$, the energy efficiency is calculated as 0.96 TOPS/1.474 W=0.65 TOPS W^-1^. And for $f_{s}$= 200 GHz, $N_{r}=9$, the energy efficiency is calculated as 32.4 TOPS/4.206 W=7.70 TOPS W^-1^.

Fig. S12 compares system power efficiency between the current volatile phase tuning schemes used in our experiment and those incorporating non-volatile PCMs versus channel size and clock frequency. The results indicate that phase tuning combined with PCMs exhibits a more rapid growth rate of system energy efficiency with increasing clock frequency and computational size, as compared to the current volatile phase tuning scheme.


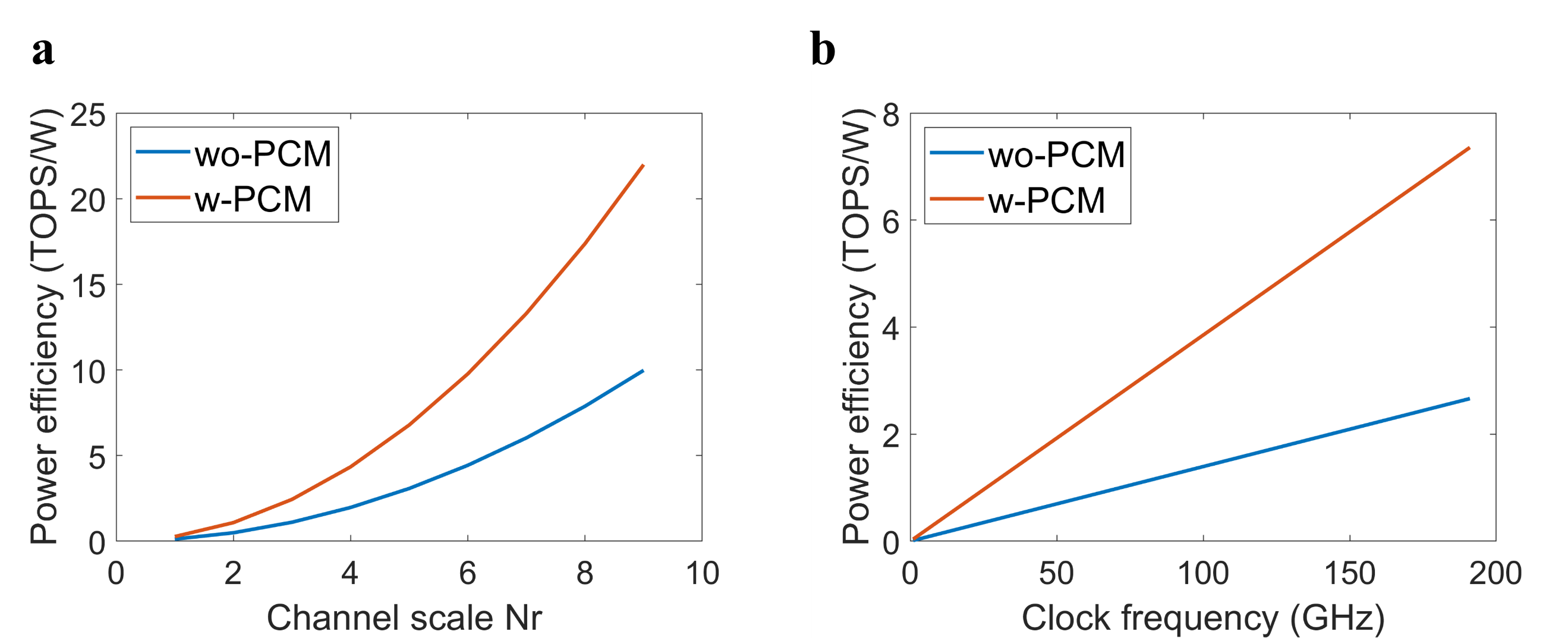


**Fig. S12 System power efficiency comparison between the current volatile phase tuning scheme and non-volatile scheme combined with PCMs.** **a.** Fixed clock frequency of 200 GHz, scanning channel size. **b.** Fixed channel size 9×9, scanning clock frequency.

A performance comparison with state-of-the-art optical and electrical approaches is provided in Table. S2. Our scheme demonstrates competitiveness in terms of kernel convolution size and system processing speed. Furthermore, by integrating non-volatile phase-change materials, the energy efficiency can be further improved.

Table. S2 Performance comparison with the-state-of-the-art works

| Year | Technology | Conv size^a^ | Data rate (GSa s^-1^) | System processing speed (TOPS) | Energy efficiency*  (TOPS W^-1^) | Ref |
| --- | --- | --- | --- | --- | --- | --- |
| 2020 | Memristor | / | / | 0.0819 | 11.014 | ^17^ |
| 2021 | Google TPUv5e | / | / | / | 1.62 | ^18^ |
| 2021 | MZI | 4 | 1×10^-5^ | 6.4×10^-7^ | 5.72×10^-7^ | ^19^ |
| 2021 | PCM crossbar | 9 | 2 | 0.256 | 0.4 | ^20^ |
| 2022 | NVIDIA H100 PCIe | / | / | / | 0.15 | ^21^ |
| 2023 | MRRs+FDL | 4 | 17 | 0.136 | 0.2 | ^22^ |
| 2023 | MRRs | 4 | 19.2 | / | / | ^23^ |
| 2024 | PCM crossbar+RF | 3 | 25 ^b^ | / | 0.54 ^b^ | ^24^ |
| 2024 | Partially coherent system | 9 | 2 | 0.108 | 1 ^b^ | ^25^ |
| 2025 | MRRs+MZI | 8 | <1×10^-6^ | / | / | ^26^ |
| 2025 | MRRs+MZI | 8 | 10 ^b^ | 2.56 ^b^ | 0.014 ^b^ | ^26^ |
| 2025 | MRRs+OTDLs | 16 | 30 | 0.96 | 0.3 | This work |
| 2025 | MRRs+OTDLs | 16 | 30 | 0.96 | 0.65 (PCM) ^b^ | This work |

FDL: fixed delay lines; PCM: phase change material; OTDL: optical tunable delay line

^a^ Conv size indicates the size of each kernel convolution

^b^ estimated

# Supplementary note 8: Insertion loss of the 3D-TPE

In the experiment architecture, insertion loss mainly comes from the following parts: (ⅰ) dual-coupled-MRRs based OCU chip; (ⅱ) optical tunable delay line-based OMU chip; (ⅲ) high-speed modulator; (ⅳ) WDM and wave-shaper; (v) optical fibers and connectors, etc.

The dual-coupled-MRRs based OCU chip and the optical tunable delay line-based OMU chip were packaged with polarization-maintaining fiber arrays, with insertion losses of approximately 7 dB and 8 dB (including 2dB per facet of coupling loss), respectively. The high-speed modulator used in the experiment is a commercial modulator with a bandwidth of 40 GHz and an insertion loss of about 4 dB. The four-channel WDM has an insertion loss of about 1 dB, and the wave-shaper has an insertion loss of about 6 dB. To confirm the polarization state of the input optical signals, two fiber polarization couplers (FPCs) were used per channel, each with a loss of about 0.5 dB. Finally, the fiber connections between individual components contribute a total insertion loss of about 2 dB. The specific loss of each part is listed in the Table. S3. For a single computation channel, the total insertion loss is calculated to be 0.5×2+1+4+6+8+7+2=29 dB. To compensate for the link loss, an erbium-doped fiber amplifier (EDFA) was employed following the modulator. The non-uniformity of optical amplification across different wavelengths, and the amplified spontaneous emission (ASE) noise of the EDFA, deteriorate the signal-to-noise ratio and increase the computational errors. Since the majority of losses occur in the fiber-chip coupling and polarization controlling, through monolithic integration and further optimization of devices, the overall insertion loss can be reduced to below 15 dB in the future. It is anticipated that eliminating the EDFA is expected to further improve signal quality and reduce computational errors.

Table. S3. Insertion loss of the devices

| Item | Insertion loss(dB) |
| --- | --- |
| FPC | $0.5\times2$ |
| WDM | 1 |
| Modulator | 4 |
| Wave-shaper | 6 |
| OMU chip | 4(on-chip)+4(CL) |
| OCU chip | 3(on-chip)+4(CL) |
| Fiber connectors | 2 |

^CL: coupling loss^

# Supplementary note 9: Compute density evaluation

Compute density is calculated to evaluate the throughput performance of the proposed 3D-TPE:

Compute Density$=\frac{Tera Operations Per Second(TOPS)}{Chip Area({mm}^{2})}$

The OCU chip and the OMU chip used in our experiment have footprints of $3.17 \mathrm{mm}\times2 \mathrm{mm}$ and $10.9 \mathrm{mm}\times3.76 mm$, respectively. The minimal chip area to fit both OMU and OCU on the same chip (eliminate the edge coupler areas for connecting chips) is estimated to be $3.5\times(10+1.2)=39.2\mathrm{mm}^{2}$. For a clock frequency of 30 GHz in our experiment, the throughput of $30\times4\times4\times2 \mathrm{GOPS}=$0.96 TOPS have been achieved. The compute density is estimated to be 0.96/6.34=0.1514 TOPS mm^-2^ for only the computing core and 0.96/39.2=0.0245 TOPS mm^-2^for the overall scheme.

With increased clock frequencies, the compute density can be further increased.

| Clock frequency (GHz) | OCU | OMU (bits) | Computational density (TOPS mm^-2^) | Reference |
| --- | --- | --- | --- | --- |
| 30 | $4\times4$ | 6 | ${0.1514}^{a}$/${0.0245}^{b}$ | This work |
| 50 | $4\times4$ | 6 | ${0.2524}^{a}$/${0.0408}^{b}$ | Theoretically |
| 200 | $4\times4$ | 6 | ${1.01}^{a}$/${0.1611}^{b}$ | Theoretically |

${}^{a}$ only the photonic computing unit is considered.

${}^{b}$ the overall scheme.

# Supplementary note 10: Scalability of the system

The scalability limitations of our proposed scheme focus on three main factors: the number of WEs within the OCU chip, the number of cascaded MZI switches in OMU chip, the insertion loss of the system.

For OCU chip based on micro-ring resonator (MRR) weight banks, the operating weighting channels are restricted within a single free spectral range (FSR). The maximum number of WEs in a FSR can be calculated as: $N_{r}=floor(\frac{FSR}{\lambda_{ch}})$, where $\lambda_{ch}$ is the channel spacing between WEs, $floor\left( . \right)$denotes the floor operation. Utilizing optimized multimode curved waveguides, a wide range FSR of 37 nm has been reported on the silicon platform^27^. We can reasonably assume that an FSR of about ~9 nm could be achieved on the multilayer Si_3_N_4_-on-SOI platform. In this case, the number of WEs supported in a FSR can be calculated as: $N_{r}=floor\left( \frac{FSR}{\lambda_{ch}} \right)=floor\left( \frac{9}{0.8} \right)=11$ for a channel spacing of 100 GHz.

The maximum on-chip insertion loss (excluding the edge coupling loss) of wavelength-routed crossbar structured OCU chip with a size of $N_{r}\times N_{r}$ is expressed as:

$${IL}_{MRR}={IL}_{drop}+ \left( N_{r}-1 \right)\times{(IL}_{Si\_through}+{IL}_{SiN\_through})+2\times\left( N_{r}-1 \right)\times{IL}_{crossing}+{IL}_{path}+{IL}_{Si\_SiN}$$

where ${IL}_{drop}$ is the drop-port insertion loss at resonant WE, ${IL}_{Si\_through}$ and ${IL}_{SiN\_through}$ are the through-pass insertion loss at non-resonant WEs for Si waveguide and Si_3_N_4_ waveguide transmission directions, respectively, ${IL}_{crossing}$ is the insertion loss of 3D waveguide crossing, ${IL}_{path}$ is the propagation loss of the connecting waveguide, and ${IL}_{Si\_SiN}$ is the interlayer coupling loss between the top Si_3_N_4_ waveguide and the bottom Si waveguide, only one used per weighting path. According to our previous measurement results ^28,29^, ${IL}_{drop}$ is ~0.4 dB, ${IL}_{Si\_through}$ and ${IL}_{SiN\_through}$ are approximately ~0.021 dB and ~0.07 dB, respectively, ${IL}_{crossing}$ is approximately 5.5×10^-3^ dB, and ${IL}_{Si\_SiN}$ is about 0.15 dB, respectively. ${IL}_{path}$ is omitted for simplicity. To be noted, the OCU chip used in this work consists of two polarization beam splitter and rotator (PSRs) in each path^28^, which introduces an additional loss of ~1 dB. The PSRs can be removed for the 3D-TPE application.

The transmission spectral characteristics across multiple resonance orders were also investigated. Fig. S13a shows the measured transmission spectra (including the edge-coupling losses) of four WEs within the OCU chip in a wavelength range of 20 nm centered at 1550 nm. The insertion losses at the resonant wavelengths across five resonance orders are illustrated in Fig. S13b. The maximum loss difference across resonance orders for four WEs are 0.088 dB, 0.115 dB, 0.25 dB, 0.199 dB, respectively. The insertion loss variation between different WEs is approximately 1 dB, primarily attributed to variations in edge-coupling losses. Benefiting from the high fabrication tolerance of the multilayer Si_3_N_4_-on-SOI platform, the OCU chip presents stable loss uniformity over a wide wavelength range.


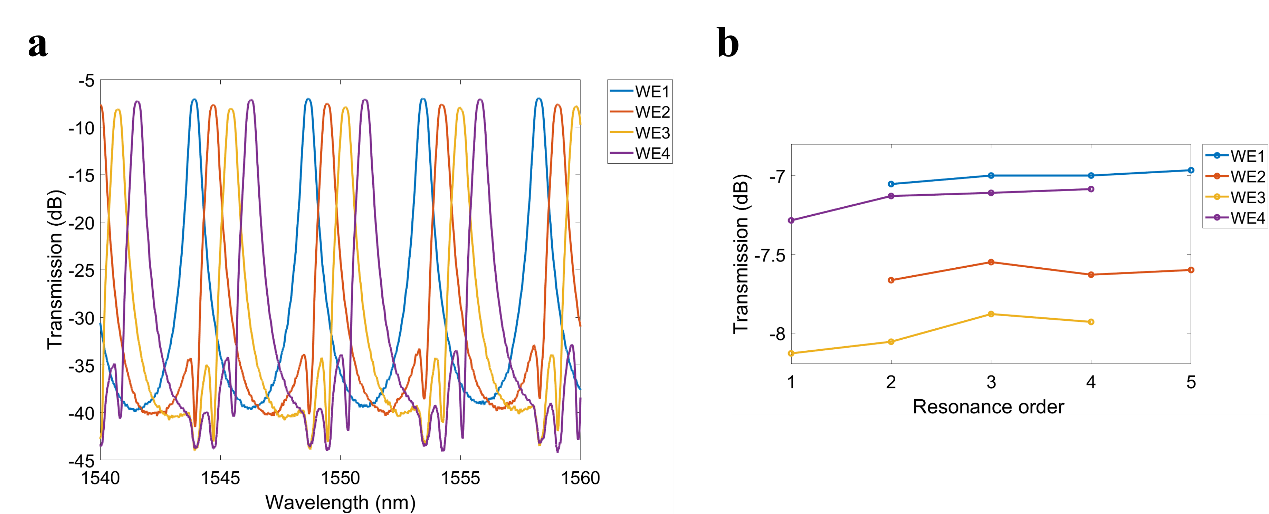


**Fig. S13 a** Measured transmission spectra (including edge-coupling losses) of four WEs within the OCU chip. **b** Insertion loss of resonant wavelengths at different resonance orders

The OMU chip is composed of optical tunable delay lines (OTDLs) based on cascaded MZI switches and delay waveguides. The maximum delay time of a OTDL is calculated as $2^{N_{mzi}-1}\delta t$, where $\delta t$ represents the delay resolution and $N_{mzi}$ presents the number of MZI switches within an OTDL. In this work, $\delta t$ is ~5 ps, achieving a balance between the reconfigurability and circuit complexity. To support the computational scale of ${N_{r}}^{2}$ on the OCU chip operating at a clock frequency of $\Delta f=1/\Delta t$, the delay time of the OTDLs within the two OMUs ranges from 0 to (${N_{r}}^{2}-1$) $\Delta t$, where $\Delta t$ is the delay time intervals between computing channels.

For the OTDLs placed at the input port of the OCU chip (marked as OMU-1), the delay time ranges from 0 to ($N_{r}-1$) $\Delta t$ with a time interval of $\Delta t$, the number of MZI switches is determined by the following equation:

${N_{mzi}=ceil(log}_{2}(\frac{(N_{r}-1)\times(\frac{1}{\Delta f})}{t})$+1)

where the $ceil(.)$ is the upward rounding function.

For the OTDLs placed at the output port of the OCU chip (marked as OMU-2), the delay time ranges from 0 to (${N_{r}}^{2}-N_{r}$) $\Delta t$ with a time interval of $N_{r}\Delta t$. If the delay resolution of the OTDLs is designed as $N_{r}\delta t$, the required MZI number of the OTDLs is the same as those in OMU-1.

Thereby, the total on-chip insertion loss of the two OMU chips are estimated to be:

${IL}_{OMUs}$= $2\times N_{mzi}\times{IL}_{0}+({N_{r}}^{2}-1)\times(\frac{1}{\Delta f})\times{IL}_{wg}$

where ${IL}_{0}$ is the insertion loss of a basic MZI switch, which is approximately 0.5 dB. Due to the 2-μm-wide ridge waveguide design of the delay waveguide, the delay waveguide loss ${IL}_{wg}$is ~1 dB ns^-1^.

The size and insertion loss statistics of the two OMU chips for different clock frequencies and OCU chip sizes are shown in Fig. S14. For clock frequencies from 10 to 200 GHz and the number of WEs ${N_{r}}^{2}$from 4 to 121, the maximum number of the cascaded MZI switches in each OMU is 9. The total on-chip insertion loss of two OMU chips ranges from 1.015 dB to 21 dB.


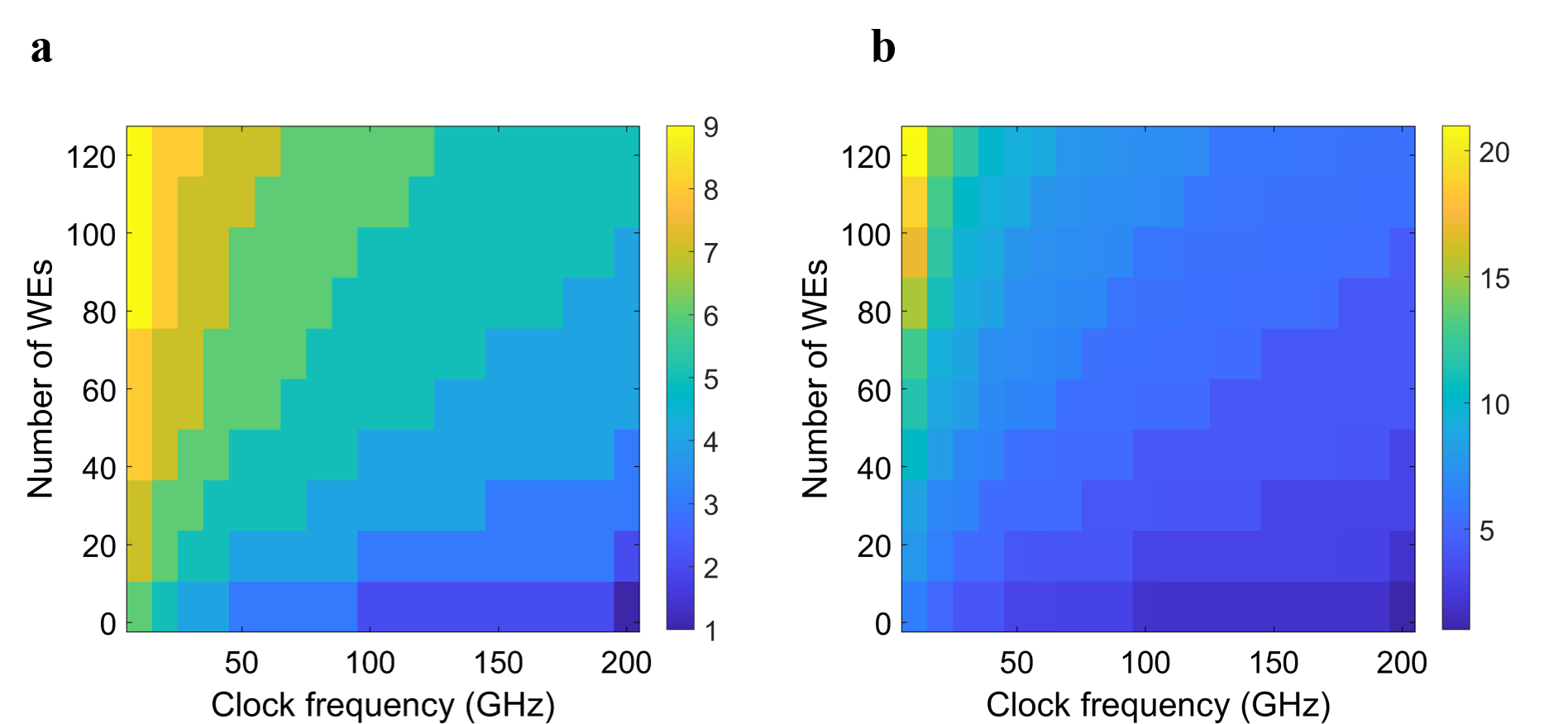


**Fig. S14 Scalability and insertion loss of OMUs versus the clock frequencies and the number of WEs in the OCU chip. a** The number of MZI switches in each OTDL. **b** Total insertion loss (excluding the edge coupling loss) of the two OMU chips.

To ensure computation accuracy, an SNR greater than 10 is considered reasonable^25^. The noise power of the TIA at 25 GHz is estimated to be –28 dBm^24^, thus the minimum required optical power before detection by PD is approximately -18 dBm. Assume a maximum optical input power of 28 dBm at the input of the high-speed modulator, the insertion loss of the integrated system must be less than 46 dB to ensure a reasonable computation accuracy. For further implementation, the OMU and OCU can be fabricated on a single chip as well as the high-speed modulator, power splitter and photodetectors, the total on-chip insertion loss of the proposed 3D-TPE are estimated by the following equation:

${IL}_{total}$ = ${IL}_{MRR}+{IL}_{OMUs}$+$abs(10\times{log}_{10}(\frac{1}{N_{r}}))+4$

where the first term is from the OCU, the second term is the total insertion loss of two OMUs, the third term is from the power splitter after the modulator, and the last term is the insertion loss of modulator. For clock frequencies from 10 to 200 GHz and the number of WEs from 4 to 121, the on-chip loss ranges from 8.64 dB to 36.95 dB, as shown in Fig. S15.


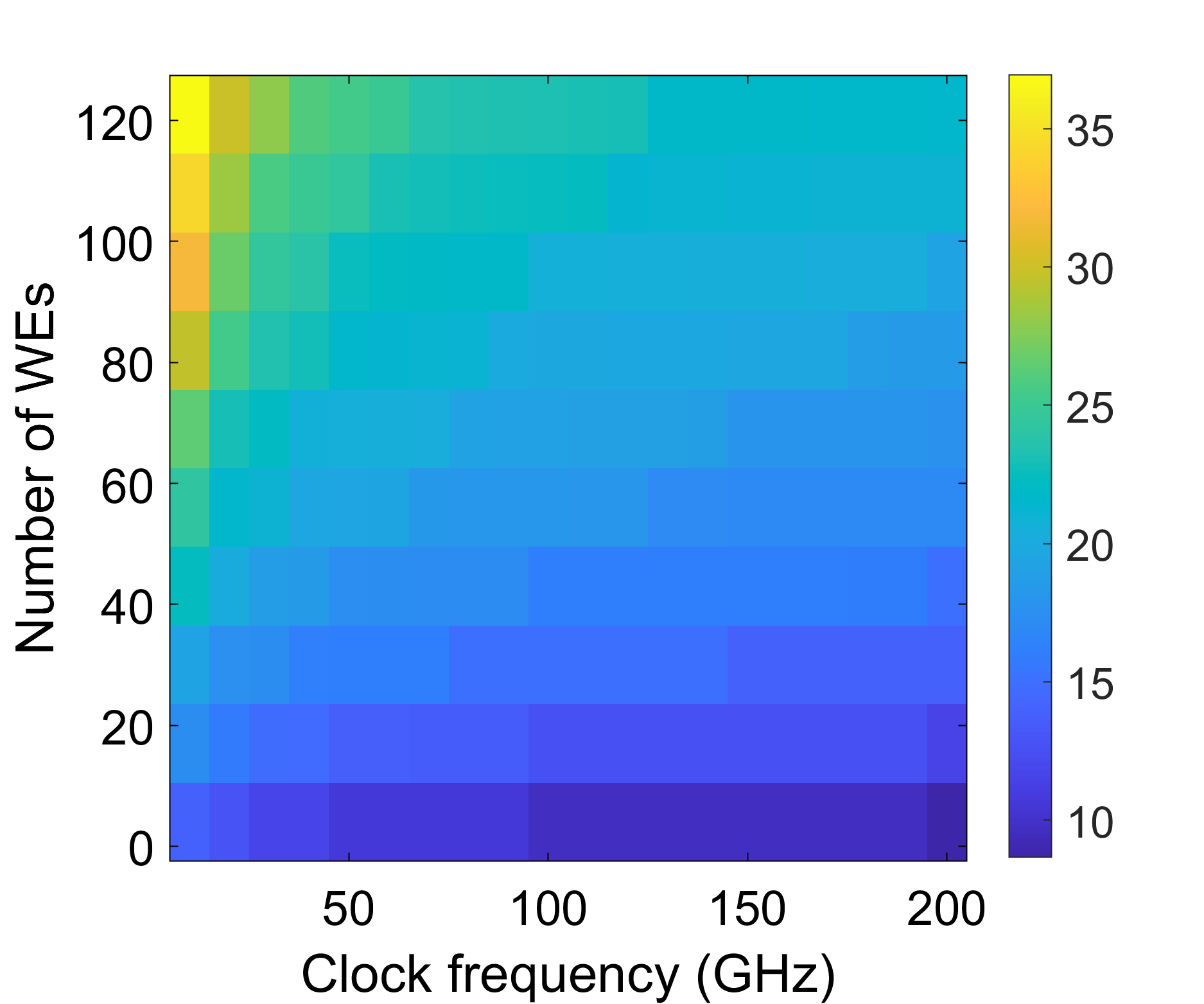


**Fig. S15** Insertion loss of the 3D-TPE versus the clock frequencies and the number of WEs.

# Reference

1. Xu, X. *et al.* Self-calibrating programmable photonic integrated circuits. *Nature Photonics* **16**, 595–602, doi: 10.1038/s41566-022-01020-z (2022).

2. Huang, C. *et al.* A silicon photonic–electronic neural network for fibre nonlinearity compensation. *Nature Electronics* **4**, 837–844, doi: 10.1038/s41928-021-00661-2 (2021).

3. Bai, B. *et al.* Microcomb-based integrated photonic processing unit. *Nature Communications* **14**, 66, doi: 10.1038/s41467-022-35506-9 (2023).

4. Xu, T. *et al.* Control-free and efficient integrated photonic neural networks via hardware-aware training and pruning. *Optica* **11**, 1039, doi: 10.1364/OPTICA.523225 (2024).

5. Ni, Z. *et al.* Silicon-Integrated 8-Channel 6-bit Tunable Optical True-Time Delay Lines with High Switching Speed and Low Loss. *2023 International Topical Meeting on Microwave Photonics (MWP)* (2023).

6. Cusmai, M. *et al.* A 0.92-pJ/b PAM-4 and 0.61-pJ/b PAM-6 224-Gb/s DAC-Based Transmitter in 3-nm FinFET. *IEEE Journal of Solid-State Circuits* , 1–12, doi: 10.1109/JSSC.2024.3456672 (2024).

7. Pfaff, D. *et al.* 7.3 A 224Gb/s 3pJ/b 40dB Insertion Loss Transceiver in 3nm FinFET CMOS. *2024 IEEE International Solid-State Circuits Conference (ISSCC)* (2024).

8. Wang, J. Q. *et al.* 7.1 A 2.69pJ/b 212Gb/s DSP-Based PAM-4 Transceiver for Optical Direct-Detect Application in 5nm FinFET. *2024 IEEE International Solid-State Circuits Conference (ISSCC)* (2024).

9. Nguyen, R. L. *et al.* 18.4 A 200GS/s 8b 20fJ/c-s Receiver with >60GHz AFE Bandwidth for 800Gb/s Optical Coherent Communications in 5nm FinFET. *2024 IEEE International Solid-State Circuits Conference (ISSCC)* (2024).

10. Khairi, A. *et al.* A 1.41-pJ/b 224-Gb/s PAM4 6-bit ADC-Based SerDes Receiver With Hybrid AFE Capable of Supporting Long Reach Channels. *IEEE Journal of Solid-State Circuits* **58**, 8–18, doi: 10.1109/JSSC.2022.3211475 (2023).

11. Kim, J. *et al.* 8.1 A 224Gb/s DAC-Based PAM-4 Transmitter with 8-Tap FFE in 10nm CMOS. *2021 IEEE International Solid- State Circuits Conference (ISSCC)* (2021).

12. Kossel, M. A. *et al.* 8.3 An 8b DAC-Based SST TX Using Metal Gate Resistors with 1.4pJ/b Efficiency at 112Gb/s PAM-4 and 8-Tap FFE in 7nm CMOS. *2021 IEEE International Solid- State Circuits Conference (ISSCC)* (2021).

13. Lin, H. *et al.* ADC-DSP-Based 10-to-112-Gb/s Multi-Standard Receiver in 7-nm FinFET. *IEEE Journal of Solid-State Circuits* **56**, 1265–1277, doi: 10.1109/JSSC.2021.3051109 (2021).

14. Li, K. *et al.* An integrated CMOS–silicon photonics transmitter with a 112 gigabaud transmission and picojoule per bit energy efficiency. *Nature Electronics* **6**, 910–921, doi: 10.1038/s41928-023-01048-1 (2023).

15. Patel, D., Sharif-Bakhtiar, A. & Carusone, T. C. A 112-Gb/s —8.2-dBm Sensitivity 4-PAM Linear TIA in 16-nm CMOS With Co-Packaged Photodiodes. *IEEE Journal of Solid-State Circuits* **58**, 771–784, doi: 10.1109/JSSC.2022.3218558 (2023).

16. Yang, X. *et al.* Non‐Volatile Optical Switch Element Enabled by Low‐Loss Phase Change Material. *Advanced Functional Materials* , 2304601, doi: 10.1002/adfm.202304601 (2023).

17. Yao, P. *et al.* Fully hardware-implemented memristor convolutional neural network. *Nature* **577**, 641–646, doi: 10.1038/s41586-020-1942-4 (2020).

18. Tensor Processing Unit. *Wikipedia* (2025).

19. Zhang, H. *et al.* An optical neural chip for implementing complex-valued neural network. *Nature Communications* **12**, 457, doi: 10.1038/s41467-020-20719-7 (2021).

20. Feldmann, J. *et al.* Parallel convolutional processing using an integrated photonic tensor core. *Nature* **589**, 52–58, doi: 10.1038/s41586-020-03070-1 (2021).

21. Xu, Z. *et al.* Large-scale photonic chiplet Taichi empowers 160-TOPS/W artificial general intelligence. *Science* **384**, 202–209, doi: 10.1126/science.adl1203 (2024).

22. Bai, B. *et al.* Microcomb-based integrated photonic processing unit. *Nature Communications* **14**, 66, doi: 10.1038/s41467-022-35506-9 (2023).

23. de Lima, T. F. *et al.* Real-Time Blind Source Separation with Integrated Photonics for Wireless Signals. (2023).

24. Dong, B. *et al.* Higher-dimensional processing using a photonic tensor core with continuous-time data. *Nature Photonics* , doi: 10.1038/s41566-023-01313-x (2023).

25. Dong, B. *et al.* Partial coherence enhances parallelized photonic computing. *Nature* **632**, 55–62, doi: 10.1038/s41586-024-07590-y (2024).

26. Wang, Y. *et al.* Reconfigurable versatile integrated photonic computing chip. *eLight* **5**, 20, doi: 10.1186/s43593-025-00098-6 (2025).

27. Liu, D. *et al.* High-Order Adiabatic Elliptical-Microring Filter with an Ultra-Large Free-Spectral-Range. *Journal of Lightwave Technology* **39**, 5910–5916, doi: 10.1109/JLT.2021.3091724 (2021).

28. Li, X. *et al.* Ultra-low-loss multi-layer 8 × 8 microring optical switch. *Photonics Research* **11**, 712, doi: 10.1364/PRJ.479499 (2023).

29. Li, X. *et al.* Low-Loss and Power-Efficient Polarization-Diversity 4 × 4 Microring Switch on a Multi-Layer Si3N4-on-SOI Platform. *Journal of Lightwave Technology* , 1–10, doi: 10.1109/JLT.2024.3449432 (2024).
